# Establishing safe high hydrostatic pressure devitalization thresholds for autologous head and neck cancer vaccination and reconstruction

**DOI:** 10.1038/s41420-023-01671-z

**Published:** 2023-10-23

**Authors:** Claudia Maletzki, Vivica Freiin Grote, Friederike Kalle, Thoralf Kleitke, Annette Zimpfer, Anne-Sophie Becker, Wendy Bergmann-Ewert, Anika Jonitz-Heincke, Rainer Bader, Brigitte Vollmar, Stephan Hackenberg, Agmal Scherzad, Robert Mlynski, Daniel Strüder

**Affiliations:** 1 Department of Internal Medicine, Medical Clinic III - Hematology, Oncology, Palliative Medicine, Rostock University Medical Center, Rostock, Germany; 2 Research Laboratory for Biomechanics and Implant Technology, Department of Orthopedics, Rostock University Medical Centre, Rostock, Germany; 3 Department of Otorhinolaryngology, Head and Neck Surgery “Otto Körner”, Rostock University Medical Center, Rostock, Germany; 4 Institute of Pathology, Rostock University Medical Center, Rostock, Germany; 5 Core Facility for Cell Sorting and Cell Analysis, University Medical Center Rostock, Rostock, Germany; 6 Institute for Experimental Surgery, Rostock University Medical Center, Rostock, Germany; 7 Department of Otorhinolaryngology-Head and Neck Surgery, RWTH Aachen University Hospital, Aachen, Germany; 8 Department of Oto-Rhino-Laryngology, Plastic, Aesthetic and Reconstructive Head and Neck Surgery, University of Wuerzburg, Wuerzburg, Germany

**Keywords:** Head and neck cancer, Cancer prevention

## Abstract

High hydrostatic pressure specifically devitalizes cells and tissues without major changes in their molecular structure. Hence, high hydrostatic pressure may enhance the development of whole-cell anti-tumor vaccines, representing tumor heterogeneity and thus (neo-) antigen diversity. Moreover, safe devitalization of tumor-infiltrated supporting tissue may facilitate reimplantation for functional reconstruction. However, precise high hydrostatic pressure thresholds for safe cancer cell killing are unknown. Here, we show that high hydrostatic pressure of at least 315 MPa is necessary to safely devitalize head and neck squamous cell cancer. A pressure of 210 MPa, which has been used frequently in cancer vaccine preparation, resulted in partial devitalization with 27% live cells in flow cytometry and 4% remaining autofluorescence in cell culture after one week. The remaining cells could form vital tumors in the chorioallantoic membrane assay. In contrast, 315 MPa killed all cells in vitro and prevented tumor outgrowth in ovo. The effectiveness of 315 MPa was attributed to the induction of DNA double-strand breaks, independent of apoptosis, autophagy, or methuosis. Furthermore, 315 MPa continued to induce immunogenic cell death. Our results demonstrate that 315 MPa of high hydrostatic pressure induces safe and sustained devitalization of head and neck cancer cells and tissues. Because of the heterogeneity in pressure resistance, we propose our approach as a starting point for determining the precise thresholds for other cancer entities. Further studies on head and neck cancer should focus on immunological co-cultures, combinations of immune checkpoint inhibition, and accurate anatomical reconstruction with pressure-treated autografts.

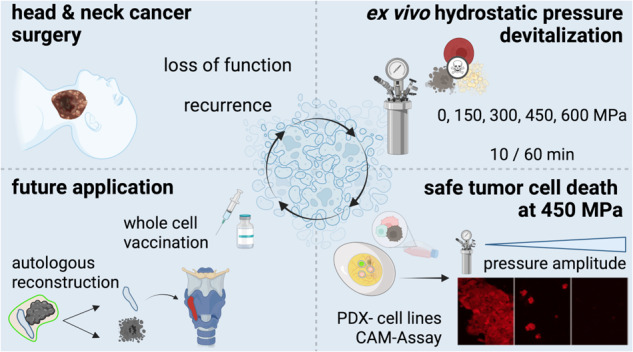

## Introduction

High hydrostatic pressure (HHP) enables complete devitalization of complex tissues of any origin without major changes in structure and biomechanics [1–9]. On the one hand, HHP triggers specific cell death by altering the plasma membrane and the tertiary/quaternary protein structure [1, 2]. In contrast, HHP preserves the biomechanics of the extracellular matrix and tumor neoantigens [2–9]. In addition, HHP treatment is cost-effective and avoids toxic substances [4]. Another advantage is the high standardization according to Pascal’s law: pressure acts isostatically and immediately at any cell within complex tissues [10]. Therefore, dose gradients and build-up effects were not observed in this study [4]. At the same time, HHP acts rapidly and can be applied to tissues ex situ during surgery.

Two significant complications in head and neck squamous cell cancer (HNSCC) are loss of function due to tissue defects and recurrence, with a low response to secondary treatment options, including immunotherapy. Therefore, head and neck surgeons can use HHP to kill all tumor cells while preserving a biomechanically stable extracellular matrix for reimplantation. In addition, the natural extracellular matrix and preserved cytokines can induce tissue-specific remodeling, proliferation, and migration into bone, cartilage, and skin. Hence, HHP may be highly beneficial for anatomical and functional reconstruction of head and neck defects [11–19].

The second application of HHP could be autologous anticancer vaccination for re-activating antigen-specific immune responses. HHP constitutes an excellent alternative to devitalization methods, such as radiotherapy, freeze-drying, and solvents [8, 20]. Indeed, dendritic cells loaded with HHP-treated tumor cells elicited specific anti-tumor immune responses in co-culture with immune cells [20–24]. Furthermore, the injection of HHP-treated melanoma cells into mice inhibited tumor growth when combined with irradiation [9, 10, 25]. Hence, HHP is promising for anatomical reconstruction using tumor-infiltrated tissue autografts and immunotherapy for restoring immunosurveillance.

A precondition for the clinical application of HHP-treated tumor cells is safe devitalization. The threshold for the complete devitalization of various cancer entities has been reported to be 250–210 MPa [4–10, 17, 20, 21, 23, 26–32]. However, most studies do not specify a threshold, leaving great uncertainty about the amplitude required to yield complete devitalization (Table 1). Owing to their immunological focus, most previous studies recommend 200 and 210 MPa to achieve an optimal balance between devitalization and preservation of the tumor-specific immune response [9, 20, 21]. However, the use of low pressure carries the risk of transplanting the remaining vital cells. Such residual cells pose a high risk of tumor recurrence; indeed, viable cells have remained in some studies. Therefore, to minimize the risk of vital tumor cell transplantation, it is necessary to define safe devitalization thresholds for each tumor entity.

**Table 1:** Cancer devitalization using high hydrostatic pressure.

Authors	Pressure amplitude [MPa]	Complete devitalization [MPa]	Time [min]	Cancer	Remaining viable cells
Rückert et al. [25]	200	N/A	5	Melanoma, mammary	N/A
Seitz et al. [10]	100–500	300	10–15	Melanoma, colon	None
Urbanova et al. [20]	150–350	200	10–15	Prostate, ovary, lung	None
Hradilova et al. [22]	250	250	10	Lung	N/A
Mikyskova et al. [21]	200	N/A	10	Lung, prostate	<10%
Chen et al. [26]	0.01–0.1	N/A	>60	Urothelial	75 × 10^4^
Fucikova et al. [23]	150–350	250	10	Leukemia, ovary prostate	N/A
Weiss et al. [4]	100–500	300	5	Melanoma, colon, lymphoma	“Few viable cells”'
Schauwecker et al. [27]	150–300	300	10	Chondrosarcoma, ostesarcoma	None
Eisenthal et al. [7]	60–120	N/A	15	Melanoma	N/A

In this study, a safe threshold for the devitalization of HNSCC was determined to enable the future application of HHP treatment in clinical trials. Commercial and patient-derived cell lines were treated at pressures between 105–420 MPa for 10–60 min. The percentage of dead cells and markers of processes involved in cell death were analyzed in vitro. In addition, engraftment was analyzed using the chorioallantoic membrane (CAM) in ovo model. Compared to previous research, a significantly higher threshold of 315 MPa was identified for safe devitalization. The application of this HHP amplitude is a precondition for the use of HHP in further studies on reconstruction and autologous anti-tumor vaccination.

## Results

### HHP treatment with 210 and 315 MPa devitalized established (UT-SCC-14) and patient-derived cell lines (HNSCC16 and HNSCC46) in the crystal violet assay

**Fig. 1:**
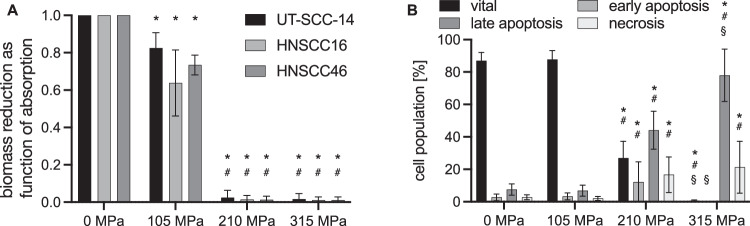
High hydrostatic pressure (HHP) leads to the devitalization of head and neck cancer cells (HNSCC). **A** Biomass reduction as function of relative crystal violet absorption measurement 72 h after treatment. HHP caused dose-dependent cell death in UT-SCC-14 and HNSCC16/46. At 105 MPa, relative absorption reduced by 18–27%. Cytotoxicity increased at 210 MPa and 315 MPa with a maximum absorption for UT-SCC-14: 2.4 ± 4% (210 MPa) and 1.6 ± 3% (315 MPa). Results shown as means and standard deviations; *n* = 5. Statistical analysis: two-way ANOVA, Tukey’s multiple comparison post hoc test; *P < 0.05, vs. 0 MPa (UT-SCC-14/HNSCC16/46); ^#^*P* < 0.05, vs. 105 MPa. **B** Cell death analysis using Yo-Pro 1 iodide/propidium iodide (Yo-Pro 1/PI) flow cytometry 24 h after treatment with UT-SCC-14. HHP at 105 MPa had no effect on vital, apoptotic, and necrotic cell populations. At 210 MPa, the number of apoptotic and necrotic cells increased significantly, but 27 ± 10% remained viable. At 315 MPa, viable cells reduced to 0.6 ± 0.5%. Values are given as means and standard deviations; *n* = 18; two-way ANOVA, Tukey’s multiple comparison post hoc test; *P < 0.05, vs. 0 MPa; ^#^*P* < 0.05, vs. 105 MPa; ^§^*P* < 0.05^,^ vs. 210 MPa.

Both cell growth (Supplementary Fig. 1) and viability (Fig. 1A) were moderately reduced following exposure to 105 MPa for 10 min; however, the cells recovered completely over time. Quantification of the viable biomass showed a reduction with 105 MPa treatment compared to 0 MPa (100%) for all three cell lines: UT-SCC-14 82 ± 8%, HNSCC16 64 ± 18%, and HNSCC46 73 ± 5%; *P* < 0.05, 105 MPa vs. 0 MPa (Fig. 1A). The absorption of viable biomass after 210 MPa was 2 ± 4% (UT-SCC-14), 1 ± 2% (HNSCC16), and 1 ± 2% (HNSCC46); *P* < 0.05, 210 MPa vs. 0 and 105 MPa. Even after the application of 315 MPa HHP, viable biomass was detected with absorptions of 2 ± 3% (UT-SCC-14), 1 ± 1% (HNSCC16), and 1 ± 1% (HNSCC46); *P* < 0.05, 315 MPa vs. 0 and 105 MPa (Fig. 1A).

### Flow cytometric apoptosis/necrosis analysis revealed complete devitalization for HHP treatment with 315 MPa, but not for 210 MPa

HHP treatment at 105 MPa had no persistent impact on viability, as compared to the 0 MPa control (87.1 ± 5.0%), and a similar percentage of vital cells (87.7 ± 5.5%) was detected after 105 MPa (Fig. 1B). In addition, no induction of apoptosis or necrosis was observed (0 MPa: early apoptosis 2.6 ± 2.1%, late apoptosis 7.5 ± 3.5%, necrosis 2.8 ± 1.5%; 105 MPa: early apoptosis 3.3 ± 2.1%, late apoptosis 6.9 ± 3.4%, necrosis 2.1 ± 1.3%). In contrast, 210 MPa induced significant apoptotic cell death, but 26.9 ± 10.4% of the cells remained viable (210 MPa: early apoptosis 12.2 ± 12.4%, late apoptosis 44.1 ± 11.7%, necrosis 16.7 ± 11.0%; *P* < 0.05, 210 MPa vs. 0 and 105 MPa). HNSCC cell treatment at 315 MPa resulted in complete devitalization. As a result of the pressure exposure, only 0.6 ± 0.5% of treated cancer cells were identified as viable (*P* < 0.05, 315 MPa vs. 0, 105, and 210 MPa). Furthermore, 12.2 ± 12.4% of the treated cells were early apoptotic (*P* < 0.05, 315 MPa vs. 210 MPa), while a late apoptotic phenotype was characterized for 44.0 ± 11.7% of the treated cells (*P* < 0.05, 315 MPa vs. 0, 105 and 210 MPa), and 16.7 ± 11.0% were necrotic (*P* < 0.05, 315 MPa vs. 0 and 105 MPa). The significance of the remaining viable cells was examined in further experiments using autofluorescent cells.

### 315 MPa HHP was the threshold for complete suppression of NIR680 autofluorescence in transfected PE/CA/PJ-15

**Fig. 2:**
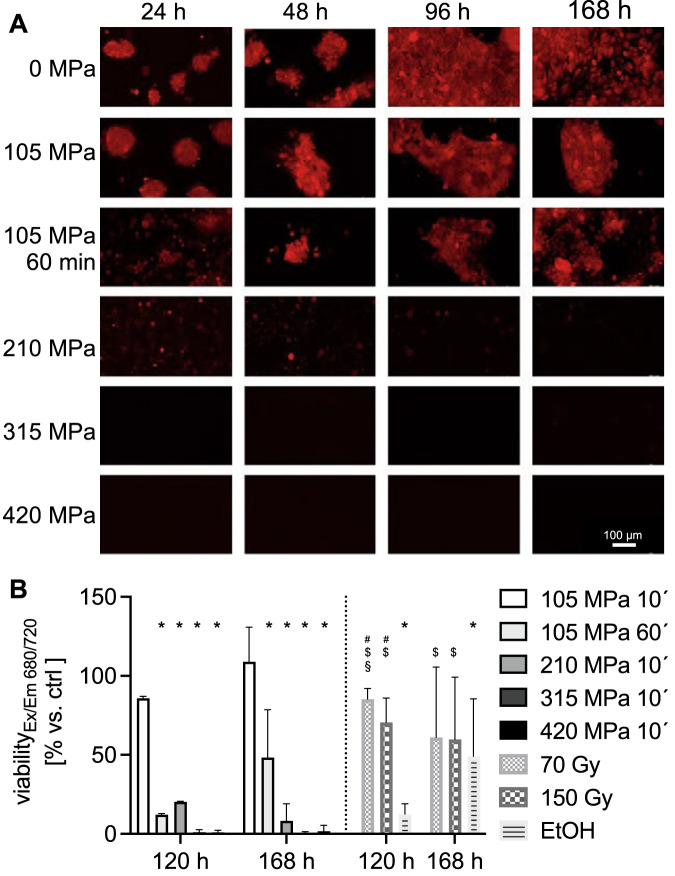
At least 315 MPa of hydrostatic pressure (HHP) is needed for the complete devitalization of PE/CA/PJ-15-NIR-680 autofluorescent head and neck cancer (HNSCC) cells. **A** Colony formation of autofluorescent HNSCC cells after HHP treatment (*n* = 3): 105 MPa treatment for 10 and 60 min delayed but did not stop colony formation. 210 MPa showed no colony formation but fluorescent cells remained for 168 h. No fluorescence occurred after 315 and 420 MPa treatment. **B** Viability measured through NIR-680 fluorescence after HHP, irradiation, and ethanol (EtOH) treatment. Relative fluorescence shown compared to 0 MPa at 120 and 168 h. Values are given as means and standard deviations; *n* = 3; two-way ANOVA, Tukey’s multiple comparison post hoc test; *P < 0.05, vs. 105 MPa 10 min; ^#^*P* < 0.05, vs. 105 MPa 60 min; ^§^*P* < 0.05, vs. 210 MPa 10 min; ^$^*P* < 0.05, vs. 315/420 MPa 10 min.

After treatment with 105 MPa for 10 min, colony formation was delayed (Fig. 2A), and relative viability decreased moderately to 85.9 ± 1.2% at 120 h post-treatment (compared to 100% fluorescence following 0 MPa, Fig. 2B). As most cells remained viable, continued proliferation led to 108.9 ± 21.9% viability at 168 h (Fig. 2B). A more extended HHP treatment significantly enhanced cell death: In cells treated with 105 MPa for 60 min, viability decreased to 12.1 ± 0.8% at 120 h (*P* < 0.05, 105 MPa, 60 min vs. 105 MPa, 10 min). However, at 168 h, cells recovered to 48.3 ± 30.2% viability (*P* < 0.05, 105 MPa, 60 min *vs*. 105 MPa, 10 min). Cytotoxicity of 210 MPa for 10 min (20.3 ± 0.3%) was slightly lower than 105 MPa for 60 min at 120 h (12.1 ± 0.8%). However, cells treated at 210 MPa did not recover over time, and viability further decreased to 8.3 ± 10.9% at 168 h (*P* < 0.05, 210 MPa, 10 min *vs*. 105 MPa, 10 min). Pressures of 315 and 420 MPa for 10 min led to a depression of autofluorescence, indicating extensive cell death. Viability decreased to 1.1 ± 1.6% following 315 MPa treatment (*P* < 0.05, 315 MPa, 10 min vs. 105 MPa, 10 min) and further decreased after 420 MPa (0.9 ± 1.3%; *P* < 0.05, 420 MPa, 10 min vs. 105 MPa, 10 min) at 120 h. Notably, no recovery was seen, and viability remained low even after 168 h of re-culture (315 MPa: 0.4 ± 1.1%, 420 MPa: 1.6 ± 3.8%; *P* < 0.05, 315/420 MPa, 10 min vs. 105 MPa, 10 min). In conclusion, at least 315 MPa was necessary for all-out HNSCC cell death in vitro.

### Devitalization by HHP was more effective than high irradiation doses (75 and 150 Gy) and ethanol treatment

To compare the devitalization efficiency of HHP, irradiation as a clinically established method and 70% ice-cold ethanol, which is recommended for cell killing in live/dead assays, were used as cell death controls. NIR-680 fluorescence of PE/CA-PJ15 cells was moderately reduced at 120 h after irradiation with 75 Gy (85.4 ± 6.6%) and following treatment with 150 Gy (70.6 ± 15.3%; *P* < 0.05, 75/150 Gy vs. 210, 315, 420 MPa and 105 MPa, 60 min) (Fig. 2B). At 168 h, the cells were viable, but did not proliferate. Consequently, compared to the untreated control, fluorescence decreased to 61.1 ± 44.4% after exposure to 75 Gy and 59.9 ± 39.3% after irradiation with 150 Gy (*P* > 0.05, 75/150 Gy, 168 h vs. 75/150 Gy, 120 h). At 120 h, ethanol treatment (12.6 ± 6.5%) was more effective than irradiation and as effective as 210 MPa (*P* > 0.05). However, the remaining cells continued to grow after ethanol treatment, and viability increased to 49.1 ± 36.3% at 168 h.

### 210 MPa HHP was insufficient to prevent tumor growth in the CAM assay

**Fig. 3:**
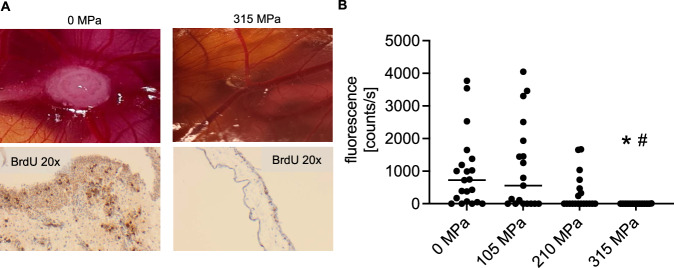
315 MPa high hydrostatic pressure (HHP) treatment prevents tumor growth on the chicken chorioallantoic membrane (CAM). **A** UT-SCC-14, HNSCC16, and HNSCC46 cells were treated with 0, 105, 210, and 315 MPa (*n* = 9–21). In all, 1 × 10^6^ cells in Matrigel were seeded on the CAM surface on embryonic development day 7. Tumors were harvested on day 14 (after 7 days of tumor growth and 1 day after topical bromodeoxyuridine (BrdU) application) for histological assessment. No tumor growth was observed after 315 MPa treatment. Representative images of HNSCC16 are shown. **B** NIR-680 fluorescence of PE/CA-PJ15 after CAM implantation at day 14 (after 7 days of tumor growth). Cancer cells could form fluorescent tumors in vivo after 105 MPa and 210 MPa treatment. Neither fluorescence nor tumor growth was observed following 315 MPa HHP. Individual values (photon counts per second (cts/s)) are plotted with median of 25% percentile/75% percentile; *n* = 19 for 105, 210, and 315 MPa; *n* = 21 for 0 MPa). Kruskal–Wallis test, Dunn’s multiple comparisons; *P < 0.05, 315 MPa vs. 0 MPa; ^#^*P* < 0.05, 315 MPa vs. 105 MPa.

To verify whether HHP-treated HNSCC cells are capable of proliferating and forming tumors in ovo, CAM assays were performed. Macroscopic and histological examination revealed that untreated HNSCC cells were able to proliferate and form tumors in ovo, while cells treated with 315 MPa showed no growth (Fig. 3A). The tumor engraftment rates of stable NIR-expressing PE/CA-PJ15 cells detected by fluorescence imaging were high at 0 MPa and 105 MPa (Fig. 3B). 0 MPa treatment led to 17 tumor engraftments out of 21 implantations. Fluorescence counts were 722 cts/s median, 25%/75%-percentile: 58/1283 cts/s. 105 MPa treatment led to 10 tumor engraftments out of 19 implantations (554 cts/s median, 25%/75%–percentile: 0/1933 cts/s). After 210 MPa treatment, moderate tumor growth was observed, and the engraftment was 7 tumor engraftments out of 19 implantations with a low fluorescence median of 0 cts/s (25%/75%-percentile: 0/470 cts/s). However, some tumors were viable with high fluorescence of 1334, 1654, and 1666 cts/s (*P* > 0.05, 210 MPa vs. 0 MPa, and 105 MPa, respectively). Finally, 315 MPa prevented tumor engraftment (0 tumor engraftments out of 19 implantations, 0 cts/s median, 25%/75%-percentile: 0/0 cts/s; *P* < 0.05, 315 MPa vs. 0 MPa and 105 MPa). One CAM exhibited a minimal fluorescence of 9 cts/s at 315 MPa. H&E histology revealed an empty Matrigel matrix without viable tumor cells (Supplementary Fig. 2). HHP at 315 MPa led to comprehensive cell death and prevented in ovo tumor growth, whereas HHP up to 210 MPa was not safe for HNSCC devitalization. There were no differences in susceptibility to pressure between the cell lines.

### HHP treatment with 315 MPa was associated with DNA double-strand breaks and induction of immunogenic cell death

**Fig. 4:**
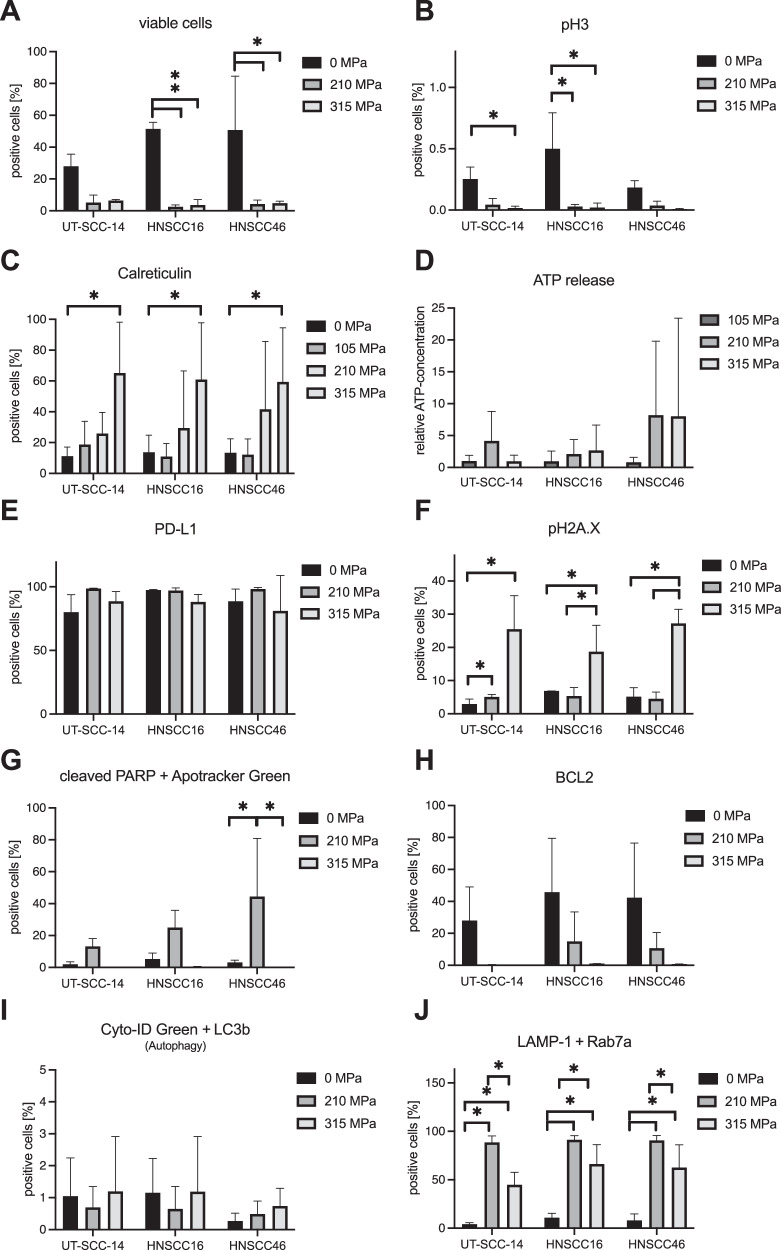
Increasing high hydrostatic pressure (HHP) induces immunogenic cell death and DNA damage in spectral multicolor flow cytometry. HHP-induced cell death was confirmed by Zombie NIR^TM^ (*n* = 3) (**A**) and inhibited cell proliferation using phosphor-histone H3 (pH3) (*n* = 3) (**B**). Immunogenic cell death was determined by an increase in calreticulin translocation (*n* = 5) (**C**) and adenosine triphosphate (ATP) release (*n* = 7) (**D**). Programmed death-ligand 1 (PD-L1) expression remained high in all groups (*n* = 3) (**E**). **F** DNA double-strand breaks, indicated by phosphorylated histone variant H2A.X (pH2A.X), were significantly increased after 315 MPa treatment (*n* = 3). Apoptosis marker cleaved poly (ADP-ribose) polymerase (PARP) was increased after 210 MPa (**G**), while anti-apoptotic marker B-cell lymphoma 2 (BCL2) decreased (**H**). After 315 MPa, all cells were negative for cleaved PARP and BCL2 (*n* = 3) (**G**, **H)**. Cyto-ID autophagy detection did not show HHP-related autophagy (*n* = 3) (**I**). Methuosis markers lysosomal-associated membrane protein 1 (LAMP-1) and Ras-related protein Rab7a (Rab7a) increased following HHP treatment. Methuosis markers were higher following 210 MPa than 315 MPa (*n* = 3) (**J**). Values are given as means and standard deviations, two-way ANOVA, Tukey’s multiple comparison post hoc test; *P < 0.05.

A comprehensive cell death panel confirmed the decreased viability and proliferation of UT-SCC-14, HNSCC16, and HNSCC46 after HHP treatment at 210 MPa and 315 MPa (*P* < 0.05, 315 MPa vs. 0 MPa) (Fig. 4A, B). Hallmarks of immunogenic cell death, such as calreticulin translocation and adenosine triphosphate (ATP) release, remained high with increased pressure amplitudes (Fig. 4C, D). Notably, calreticulin showed a pressure amplitude-dependent increase (Fig. 4C). HHP exposure had no effect on programmed death-ligand 1 (PD-L1) abundance, which remained high in all treatments (*P* > 0.05) (Fig. 4E). A significant difference between 210 and 315 MPa was found in phosphorylated histone variant H2A.X (pH2A.X) positive cells (for HNSCC16/46: *P* < 0.05, 315 MPa vs. 0 and 210 MPa), indicating DNA double-strand breaks (Fig. 4F). Considering the results of UT-SCC-14 cells, pH2A.X was detected in 2.9 ± 1.4% of the 0 MPa control cells. pH2A.X positive cells increased to 5.0 ± 0.7% after 210 MPa (*P* < 0.05, 210 MPa vs. 0 MPa). Moreover, 315 MPa HHP led to a significant increase: 25.5 ± 10.0% (*P* < 0.05, 315 MPa vs. 210 MPa). Pressure-related changes in pH2A.X were consistent in all cell lines (UT-SCC-14, HNSCC16, and HNSCC46).

**Fig. 5:**
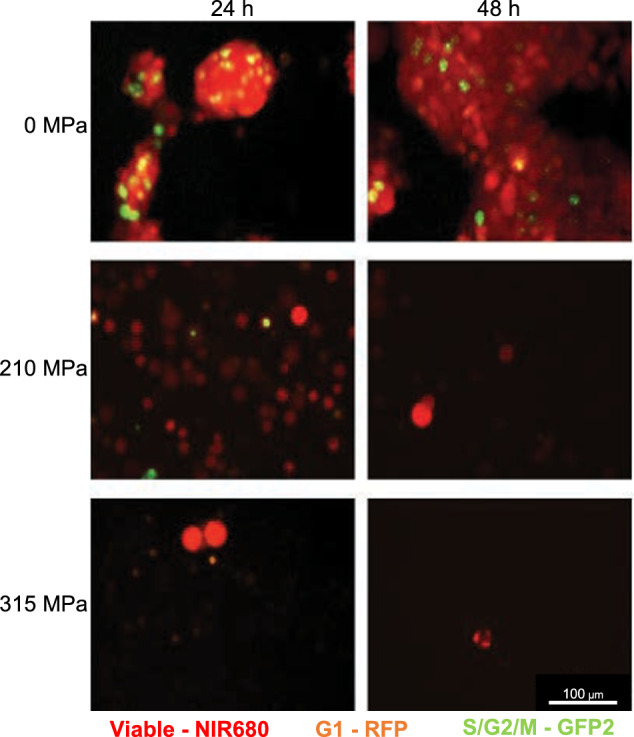
Cell cycle analysis confirmed viable cells in the S/G2/M phase following 210 MPa high hydrostatic pressure compared to 315 MPa. NIR-680 autofluorescent cells were tagged with RFP in the G1 phase and GFP2 in the S/G2/M phase. Following 210 MPa and 315 MPa treatment, viability (red) decreased over time. After 315 MPa treatment, no proliferating cells (green) were observed.

In addition, BCL2-independent and PARP-dependent apoptosis was observed after 210 MPa, but no apoptotic cells (cleaved PARP/Apotracker Green positive) were observed after 315 MPa HHP (Fig. 4G, H). Autophagy levels were not increased by HHP (*P* > 0.05), but LAMP-1/Rab7a expression as a methuosis marker was elevated at 210 MPa and 315 MPa (Fig. 4I, J). Compared to treatment with 315 MPa, 350 MPa caused significantly higher LAMP-1/Rab7a expression (*P* < 0.05) (Fig. 4J). Cell cycle analysis showed viable or proliferating cells in the G1 phase (red) and S/G2/M phases (green) 24 h after exposure to 0 and 210 MPa (Fig. 5). However, viability decreased and no proliferating cells were observed after 48 h. At 315 MPa, no cells in the S/G2/M phase were observed at 24 and 48 h.

### 315 MPa HHP devitalized human HNSCC tissue from surgical resections

**Fig. 6:**
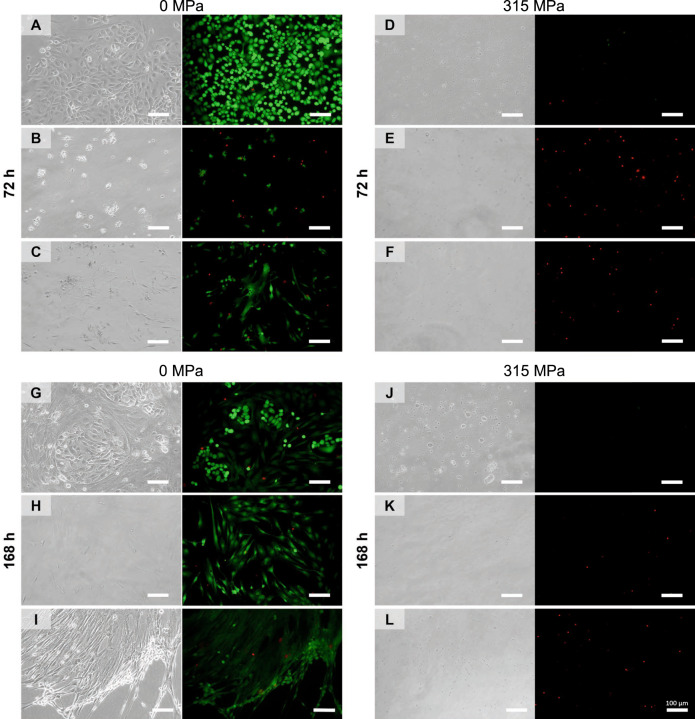
Tissue devitalization by high hydrostatic pressure (HHP). Patient-derived HNSCC tissue culture following high hydrostatic pressure treatment. Fresh Tumor samples obtained from three different patients (*n* = 3) were used to analyze tissue devitalization by HHP treatment: 88-year old female, pT4a pN0 cM0 HPV- laryngeal squamous cell cancer (**A**, **D**, **G**, **J**), 59-year old male, pT3 cN0 cM0 HPV+ laryngeal squamous cell cancer (**B**, **E**, **H**, **K**) and 55-year old male pT3 pN0 cM0 HPV- oropharyngeal squamous cell cancer (**C**, **F**, **I**, **L**). All tissue fragments were treated with either 0 MPa (**A**–**C**, **G**-**I**) or 315 MPa (**D**–**F**, **J**–**L**) for 10 min, followed by enzymatic tissue dissociation and cultivation of isolated cells. After 72 h (**A**–**F**) and 168 h (**G**–**L**), tissue devitalization was analyzed by light microscopy and live/dead staining. As a result of the treatment with 315 MPa HHP, no living cells were identified. Bar: 100 µm.

To verify whether HHP treatment can devitalize cells in the tissue association, fragments of tumor resections were investigated. The patient characteristics were as follows: An 88-year-old female with pT4a pN0 cM0 HPV^−^ laryngeal squamous cell cancer (A, D, G & J), a 59-year-old male with pT3 pN0 cM0 HPV^+^ laryngeal squamous cell cancer (B, E, H & K), and a 55-year-old male with pT3 pN0 cM0 HPV^-^ oropharyngeal squamous cell cancer (C, F, I & L). Figure 6 displays the live/dead staining and light microscopic images. Neither viable cancer cells nor viable fibroblasts were observed at 72 and 168 h, respectively. The devitalization of complex HNSCC tissues confirmed the transferability of the results at the individual cell level.

## Discussion

Devitalization is a crucial step for autologous reconstruction with tumor-infiltrated tissue and whole-tumor cell vaccination. Devitalization must be safe, induce immunogenic cell death, and comply with the legal requirements [10]. The present study aimed to establish a safe threshold for HHP treatment in HNSCC devitalization. The underlying mechanisms and immunogenic cell death were investigated using four HNSCC cell lines.

The scale and type of cell death were dependent on HHP amplitude. The cells recovered and continued proliferating at 105 MPa for 10 min and 60 min, respectively. The 60-min treatment was significantly more effective than the 10-min treatment. Reversible cell death after pressures ≤105 MPa is in line with previous research showing decent changes in membrane and protein structure [1]. In our study, high-pressure amplitudes (rather than treatment duration) were critical for irreversible devitalization; at higher pressures of 210 MPa for 10 min, the cells did not recover, and viability decreased substantially. Increasing pressure initially triggered apoptosis and, to an ascending extent, late apoptosis and necrosis. Flow cytometry confirmed a significant decrease in viable tumor cells after HHP treatment at 210 MPa; however, some cells remained alive. A minimum pressure of 315 MPa was required for sufficient cell death. In contrast to the autofluorescence measurements and flow cytometry, standard assays, such as colony formation and crystal violet staining, showed no viable cells after 210 and 315 MPa. Given that even small numbers of viable cancer cells can cause recurrence, a combination of different methods is crucial to allow reliable conclusions on the efficacy of HHP devitalization. Using a CAM assay, HNSCC cells were found to form solid tumors in ovo following treatment with 210 MPa. In contrast, no growth was observed at 315 MPa pressure.

Still, in vitro assays identified vital cell remnants. These cell remnants were reduced 10–20 times compared to those at 210 MPa. However, the importance of the few surviving cells required further investigations when evaluating oncological safety: Accompanying cell cycle analysis after HHP treatment at 210 MPa identified cells in the S/G2/M phase at 24 h. These proliferating cells could cause tumor growth under favorable in vivo conditions, such as microenvironmental niches. No S/G2/M phase labeling was observed at 315 MPa. Based on the decrease in apoptosis and methuosis at increasing pressures, it can be assumed that 315 MPa immediately causes severe cell damage. No form of programmed cell death or resilience was observed. Mechanistically, the failure to recover from 315 MPa is supported by profound DNA damage. pH2A.X DNA double-strand breaks increased significantly after 210 MPa HHP, whereas DNA damage remained low. This finding contrasts with previous studies on non-malignant cells in which DNA was reported to be the most resistant to HHP [1, 33]. DNA damage occurred only at higher pressures of >1000 MPa, whereas membrane and protein damage were critical for cell killing at pressures >200 MPa [1]. Impaired DNA repair in cancer cells could explain distinct intrinsic pressure resistance.

The effects of HHP were highly reproducible. Cell death and underlying mechanisms were consistent in commercial cell lines, patient-derived cell lines, and primary tumor tissue from surgical resections. According to Pascal’s law, the same pressure is applied at each location in an enclosed space. There are no dose gradients during HHP treatment. Each cell in suspension or tissue is exposed to a precise amplitude for a similar time. These properties lead to complete devitalization. In contrast, alternative methods, such as 75 and 150 Gy irradiation, only reduced viability, but half of the cells remained viable after irradiation. The effectiveness of HHP was also confirmed in comparison with ethanol treatment (which is recommended as a dead control in live/dead assays); ethanol was initially toxic, but the surviving cells continued to proliferate. In contrast, virtually all HHP-treated cells were devitalized.

Finally, a proof-of-concept approach was developed for complex tissues obtained from patients with HNSCC after surgery. Cell culture results were confirmed for all tumor specimens. HHP-treated HNSCC (including cartilage infiltration) did not contain any vital cells after 315 MPa in short-term culture. The devitalization and reimplantation of non-malignant complex tissues have already been tested. Subcutaneous implantation of melanocytic nevi after HHP treatment at 200 MPa resulted in fading of the nevi in a mouse model [16, 34]. Subsequently, a clinical trial was initiated to reconstruct the donor site using HHP-treated autologous nevi [18]. In orthopedics, Schauwecker et al. showed that no cells grew out of the resected tumor-infiltrated bone after 210 MPa [27, 31]. However, devitalization was only confirmed by light microscopy. Based on the present results, the threshold of 210 MPa for complete devitalization should be revised in vitro and in vivo prior to clinical application.

Most oncology studies on HHP have focused on immune activation and whole-cell vaccination using distinct HHP-treated cancer cells [4–10, 20, 21, 23, 26, 27, 29, 31]. The research groups of Adkins et al. [8, 20, 22] and Gaipl et al. [2, 4, 6, 9, 10] contributed significantly to progress in this field. However, the methodology was limited in the examination of oncological safety. In vitro methods, such as clonogenic assays, WST-8, Annexin-V/PI flow cytometry, and basic histology, were used to establish 200–210 MPa as the threshold for cellular devitalization [4, 6, 8–10, 20, 21, 23]. This threshold was supported by only one in vivo study with three mice, confirming the absence of tumor outgrowth at 200 MPa [10]. Thus, relatively low thresholds of 300 MPa maximum were defined for devitalization and preservation of immunogenicity [20]. This is in line with the findings of the clonogenic assays, Zombie staining, and crystal violet quantification. However, the critical pressure for safe devitalization was increased to 315 MPa using more sensitive assays.

Although pressure effects were highly reproducible in the four HNSCC cell lines, the pressure resistance of previously used cancer cell lines was heterogeneous. Weiss et al. reported complete devitalization of mammary adenocarcinoma (MCF7) and melanoma (B16-F10) cells by 210 MPa, whereas colon carcinoma (CT26) cells remained viable even after 500 MPa [9]. In vivo, 200 MPa was reported to be safe, and no tumor growth was observed in mice. Urbanova et al. also found that only 0.8% of viable lung cancer cells (H522 and A549) remained after 250 MPa treatment [20]. Because the release of immunogenic cytokines was higher at 200 MPa than at 250 MPa, the authors proposed lower pressures for further experiments. Given the heterogeneity of cell lines and methods, no reliable thresholds have been established to date. Nevertheless, treatment at 200–210 MPa is frequently recommended for vaccination in preclinical [4, 8–10, 20, 21, 23] and clinical trials (*NCT03657966, NCT02470468, NCT02111577*). In this study, HNSCC was more resistant to HHP, and safe devitalization was observed only at pressures above 315 MPa.

Antigen loss was previously described to be dependent on cell origin and was particularly pronounced in lung cancer but low in prostate and ovarian cancer cells [20]. Other studies have reported that the shape was maintained and immunity was preserved at pressures up to 400 MPa [2]. Although the present study focused on oncological safety, it was found that 315 MPa could still trigger immunogenic cell death. Calreticulin translocation, which is essential for phagocytosis by dendritic cells, increased in a pressure-dependent manner, with a maximum at 315 MPa. ATP release, another hallmark of immunogenic cell death, was highly variable, but increased with pressure. In addition, PD-L1 abundance remained high.

Therapeutic application of whole-tumor cells passing through immunogenic cell death leads to immune activation and exposure to a variety of tumor-specific (neo-)antigens. Especially in tumors with a high mutational burden, such as HNSCC, relevant antigens do not need to be prospectively identified. HHP-treated tumor cells can be applied directly or used to load dendritic cells [4, 20]. With direct subcutaneous administration in mice, colon (CT26) and lung (LL2) cancer cells were able to elicit CD4^+^ and CD8^+^ T cell-dependent protective immunity [35]. In other tumor entities, co-treatment is necessary to bypass immune evasion. Concurrent radiotherapy, anti-CTLA4/PD1 immunotherapy, and immunoadjuvants showed promising additive effects in vivo [25]. Dendritic cells loaded with HHP tumor cells secrete proinflammatory cytokines and stimulate IFN-γ-producing tumor antigen-specific CD4^+^ and CD8^+^ T cells in non-small cell lung cancer [22].

The second indication for HHP is autologous reconstruction in oncological head and neck surgery. Invasive tumors of the auricle, nose, and larynx lead to defects in cartilaginous support due to infiltration and resection margins [36–39]. Only a few experimental studies have been conducted on autologous laryngeal reconstruction [40–42]. However, the complex requirements of form, function, and immunosuppression (in oncologic patients) forestall allogeneic or xenogeneic cartilage transplantation in humans. Therefore, autologous laryngeal reconstruction is an attractive option with a defined clinical need, success in a structurally related organ (trachea), and no need for immediate perfusion [14, 43]. For this purpose, HHP treatment could guarantee the devitalization of all tumor cells without permanently damaging the cartilage matrix.

This study comprehensively investigated cell death in HNSCC; however, no other tumor entities have been studied. Previous research has shown a heterogeneous response between tumor entities. Owing to this possible heterogeneity in intrinsic pressure resistance, devitalization thresholds should be determined separately for each tumor entity before clinical application. While previous studies have focused on the immunogenicity of HHP-treated tumors, this study addressed safety. Methodologically, our immunological studies only provided indications of persistent immunogenic cell death at pressures above 315 MPa. Follow-up studies are needed to investigate cytokine secretion and T-cell activation in co-culture experiments. Another limitation of this study is the lack of an in vivo study using a small animal model. The CAM assay is well suited to test tumor viability by fluorescence measurement and histology, with a low experimental animal burden and high growth rates. However, chicken is evolutionarily distant from mammals; moreover, the tumor grows on an extracorporeal membrane. Therefore, invasively growing (cartilage infiltrating) tumors should be generated, HHP-treated, and reimplanted in rodents before clinical application.

### Conclusion

High hydrostatic pressure is suitable for the safe devitalization of head and neck cancer. Compared with previous studies on other entities using up to 210 MPa, higher hydrostatic pressures of more than 315 MPa are required to devitalize head and neck cancer safely. A significant difference between 210 and 315 MPa was observed in the induction of DNA double-strand breaks. In addition, immunogenic cell death accelerated with increasing pressure amplitude. These results demonstrate that HHP is a safe and reliable technology to complement autologous head and neck reconstruction and whole-cell anti-tumor immunotherapy for high mutational burden cancer.

## Materials and methods

The full methods are described in the supplemental material (see Supplemental Methods) and are summarized herein.

### Cell lines

HNSCC cells from the tongue (UT-SCC-14, PE/CA-PJ15), larynx (PDX-derived HNSCC16) [44], and hypopharynx (PDX-derived HNSCC46) [44] were used. The PE/CA-PJ15 cells were transduced to stably express iRFP680. All the cell lines were HPV-negative.

### High hydrostatic pressure treatment

Cells were suspended in the culture medium and added to cryotubes for HHP treatment. Cryotubes were filled with medium, closed without air bubbles, sealed with Parafilm, and placed in water-filled centrifuge tubes. The samples were treated for 10 or 60 min at high hydrostatic pressures (Dustec Hochdrucktechnik GmbH, Germany) of 0, 105, 210, 315, or 420 MPa. The temperature was maintained at 20 °C. Cell pellets were resuspended after treatment.

### Crystal violet assay for determination of cell viability

UT-SCC-14 and PDX-derived cell lines (*n* = 5) were incubated after HHP treatment and stained with 0.2% crystal violet for 10 min. The stained cells were washed with PBS, 1% SDS was added, and incubated for 10 min. Absorbance was measured at 560 nm using a spectrophotometer after 72 h.

### Apoptosis/necrosis and calreticulin flow cytometry

Apoptosis/necrosis was assessed 24 h after HHP treatment using a flow cytometer. UT-SCC-14 cells (*n* = 15) were stained with Yo-Pro 1 iodide (Yo-Pro 1) and propidium iodide (PI). For calreticulin (CalR) analysis, UT-SCC-14, HNSCC16 and HNSCC46 cells (*n* = 5) were incubated with primary antibody, labeled with FITC-conjugated secondary antibody, and analyzed by subtracting the secondary antibody-positive cells from CalR+ and secondary antibody-positive cells.

### Autofluorescence measurement

The autofluorescence of PE/CA-PJ15-NIR-680 cells was measured after HHP, irradiation, or ethanol treatment using a fluorescence multi-well plate reader (Tecan Infinite® M200, Germany) at 680 nm excitation and 720 nm emission wavelengths. The fluorescence intensities were calculated by setting the untreated cells at 100%. Data from at least three independent experiments performed in duplicate are presented.

### Spectral flow cytometry

This study utilized spectral flow cytometry with two custom-designed multicolor panels for functional analysis of non-transduced HNSCC cells (*n* = 3). Panel 1 was used to investigate apoptosis, necrosis, proliferation, and autophagy, while panel 2 was used to examine viability, methuosis, and immune regulation. To perform the analysis, 0.5 × 10^6^ cells were taken per panel and processed using staining buffer (PBS, 2 mM EDTA, 2% BSA).

In Panel 1, extracellular staining was conducted using Apotracker green, Mitospy^TM^ Orange CMTMRos, and Cyto-ID autophagy detection kit 2.0, and intracellular staining was performed using V450 rat anti-histone H3, Alexa Fluor 647 rabbit-anti-human light chain 3 B (LC3B), Alexa Fluor 700 mouse-anti-human cleaved poly (ADP-ribose) polymerase (PARP), and PE/Cyanine7 mouse anti-H2A.X phospho. In Panel 2, viability was assessed using Zombie NIR^TM^, and extracellular staining was conducted using PE-Vio 770 REA anti-human CD274, FITC mouse anti-human CD107a (lysosomal-associated membrane protein 1 (LAMP-1)), and Alexa Fluor 594 rat anti-human Ras-related protein Rab7a (Rab7a). Intracellular staining was performed using APC REA anti-human B-cell lymphoma 2 (BCL2) and Alexa Fluor 405 mouse anti-human Hif1α. After staining, the cells were washed and treated with the True Nuclear Transcription Factor Buffer Set for membrane permeabilization and intracellular staining. The associated flow cytometry gating strategy is shown in Supplemental Fig. 3.

### Chorioallantoic membrane assay

**Fig. 7:**
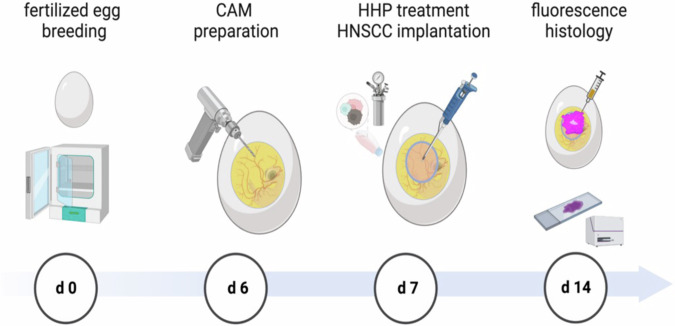
Chorioallantoic membrane (CAM) assay experimental design. Breeding of the fertilized eggs with automatic rotation started from embryonic development day zero (d 0). Eggs were opened and the CAM was prepared at day 6. On day 7, 1 × 10^6^ cells of the respective head and neck squamous cell carcinoma (HNSCC) cell line were treated with 0, 105, 210, and 315 MPa high hydrostatic pressure (HHP) at 20 °C for 10 min. Following 7 days of incubation and tumor growth, euthanasia and harvesting of the CAM were performed. Near-infrared (NIR) fluorescence was measured, and the CAM tissue was prepared for histology.

Animal procedures were conducted in accordance with national regulations (TierSchG, Germany). Lohmann Deutschland GmbH & Co. KG provided fertilized eggs, which were incubated for six days. The CAM was dropped, and a 1 cm diameter window was opened under sterile conditions [45]. The window was sealed with wound dressing, and the eggs were incubated without rotation. On day 7, the pellet of 1 × 10^6^ HHP-treated or untreated cancer cells was suspended in 20 µL Matrigel and pipetted onto the CAM. Embryos were monitored daily for one week, euthanized, and CAM tissue was harvested for ex vivo fluorescence measurements and histology. CAMs were resected and measured using a NightOWL LB 983 imaging system (Berthold Technologies, Germany) with a 630-nm excitation filter and a 700-nm emission filter (PE/CA-PJ15; *n* = 19 for 105, 210 and 315 MPa; *n* = 21 for 0 MPa). The sample size was calculated using G-Power software (HHU, Germany). Based on the in vitro experiments, the effect size was estimated to be high with Cohen η² = 0.14/ f = 0.403. Photon counts per second (cts/s) were analyzed using Indigo Software. The CAM tissue was embedded in paraffin after fixation in 4% phosphate-buffered formalin for three days. Paraffin-embedded tissue blocks were cut into 4 μm sections and stained with hematoxylin and eosin (H&E). For the experimental groups with non-fluorescent PDX-derived cell lines, 100 µL BrdU (20 mg/mL) was topically administered onto the CAM membrane 24 h before euthanasia to assess tumor tissue viability (*n* = 9–21). BrdU immunohistochemistry was performed using the DAB method, with mouse anti-BrdU as the primary antibody and goat anti-mouse HRP as the secondary antibody. The histology was analyzed by a clinical pathologist Fig. 7.

### Patient-derived HNSCC tissue culture and live/dead staining

To study the effect of HHP treatment on tumor tissue, tumor samples were obtained during surgery (Ethics Committee reference number A2018-000). After dissection, the samples were sent to the Institute of Pathology, Rostock University Medical Center, for instantaneous H&E staining. The pathologist removed the tumor tissue for routine diagnosis and provided macroscopically vital tumor tissue. Fresh tumor samples (n = 3) were then treated with 0 or 315 MPa for 10 min in air-free cryotubes within 90 min of resection. Next, the tumor tissue was dissociated using 2 mg/mL collagenase A at 37 °C under constant agitation for 2 h. The cell suspension was filtered and washed before resuspension in the cell culture medium before seeding into 12-well cell culture plates. Live/dead staining was performed after 72 or 168 h (LIVE/DEAD^TM^ Viability/Cytotoxicity Kit).

### Statistics

All values are given as mean ± standard deviation (in vitro) or as individual values with a median of 25% percentile/75% percentile (CAM assay). Statistical evaluation was performed using GraphPad PRISM software, version 8.0.2 (GraphPad Software, USA). The criterion for significance was set at *P* < 0.05. After proving the assumption of normality (Shapiro–Wilk test), two-way ANOVA with Tukey’s multiple comparison post hoc test was performed. If the normality test failed, the Kruskal–Wallis test with Dunn’s multiple comparison test was performed.

## Supplementary information

Supplemental Material

Supplemental Figure 1

Supplemental Figure 2

Supplemental Figure 3

## Data Availability

The datasets generated for this study are available on request to the corresponding author.
