# Author Correction: Establishing safe high hydrostatic pressure devitalization thresholds for autologous head and neck cancer vaccination and reconstruction

**DOI:** 10.1038/s41420-024-02172-3

**Published:** 2024-10-15

**Authors:** Claudia Maletzki, Vivica Freiin Grote, Friederike Kalle, Thoralf Kleitke, Annette Zimpfer, Anne-Sophie Becker, Wendy Bergmann-Ewert, Anika Jonitz-Heincke, Rainer Bader, Brigitte Vollmar, Stephan Hackenberg, Agmal Scherzad, Robert Mlynski, Daniel Strüder

**Affiliations:** 1 Department of Internal Medicine, Medical Clinic III - Hematology, Oncology, Palliative Medicine, Rostock University Medical Center, Rostock, Germany; 2 Research Laboratory for Biomechanics and Implant Technology, Department of Orthopedics, Rostock University Medical Centre, Rostock, Germany; 3 Department of Otorhinolaryngology, Head and Neck Surgery “Otto Körner”, Rostock University Medical Center, Rostock, Germany; 4 Institute of Pathology, Rostock University Medical Center, Rostock, Germany; 5 Core Facility for Cell Sorting and Cell Analysis, University Medical Center Rostock, Rostock, Germany; 6 Institute for Experimental Surgery, Rostock University Medical Center, Rostock, Germany; 7 Department of Otorhinolaryngology-Head and Neck Surgery, RWTH Aachen University Hospital, Aachen, Germany; 8 Department of Oto-Rhino-Laryngology, Plastic, Aesthetic and Reconstructive Head and Neck Surgery, University of Wuerzburg, Wuerzburg, Germany

**Keywords:** Head and neck cancer, Cancer prevention

Correction to: *Cell Death Discovery* 10.1038/s41420-023-01671-z, published online 23 October 2023

A correction is required to prevent subsequent studies from using higher pressures than necessary, which could have significant disadvantages in both vaccination and reconstruction applications.

Firstly, in the context of autologous head and neck cancer vaccination, applying higher pressures than necessary might alter important antigens, reducing the specificity and efficacy of the vaccine. High pressures could denature or modify antigenic proteins, compromising the vaccine’s ability to elicit a targeted immune response.

Secondly, in tissue reconstruction, the integrity of the extracellular matrix is crucial for successful tissue-specific revitalization. Excessive pressure could damage the matrix structure, undermining the primary advantage of hydrostatic high pressure treatment over other methods.

Maintaining matrix integrity is essential for the successful integration and functionality of the revitalized tissue. Furthermore, correcting the pressure values to reflect the true, lower required pressures has practical benefits. Lower pressure requirements could allow for the use of more cost-effective high-pressure reactors, facilitating broader adoption of this technology.

This would make the treatment more accessible and economically viable for a wider range of applications. By issuing this correction, we aim to ensure that future studies use the appropriate pressure values, thereby preserving the advantages of hydrostatic high pressure treatment and preventing potential negative outcomes associated with the application of unnecessarily high pressures.

Due to the systematic error, various parts of the manuscript are affected in the Abstract, Methods, Results section, Discussion section, and Figs. 1, 2, 3, 4, 5, 6.

**Fig. 1:**
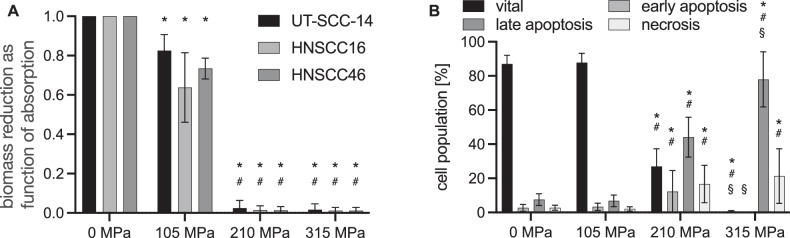
▓.

**Fig. 2:**
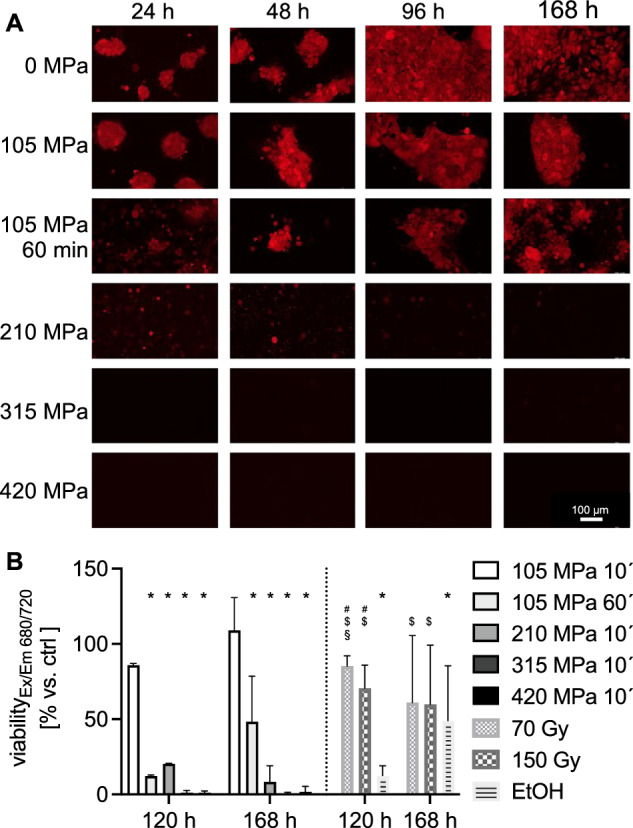
▓.

**Fig. 3:**
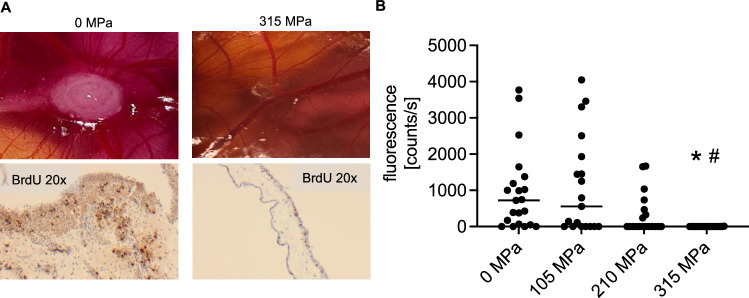
▓.

**Fig. 4:**
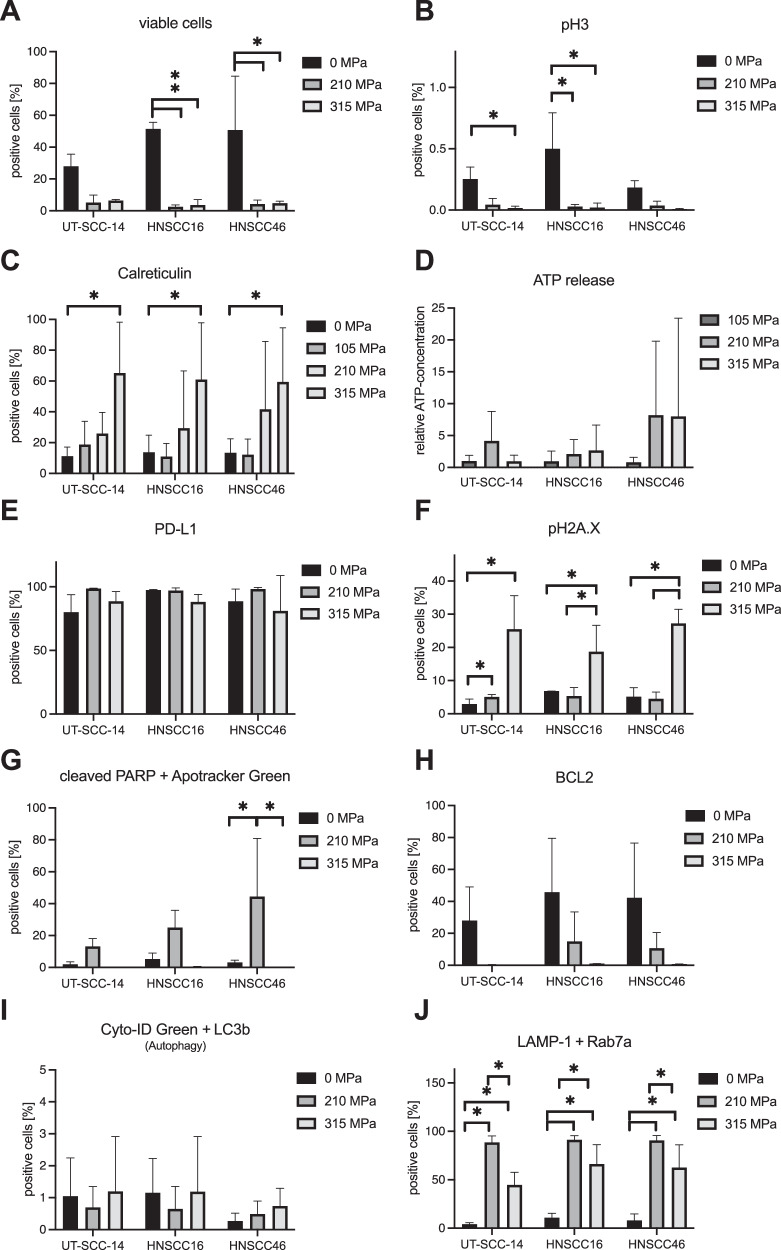
▓.

**Fig. 5:**
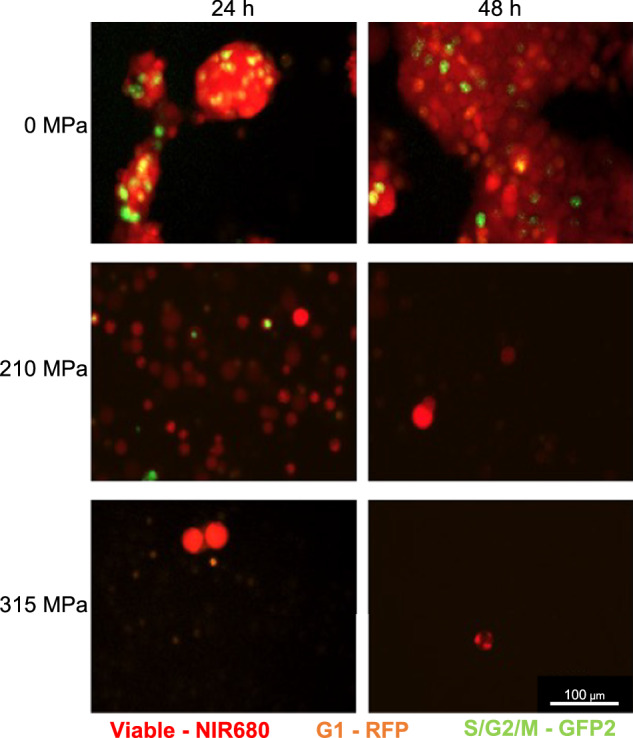
▓.

**Fig. 6:**
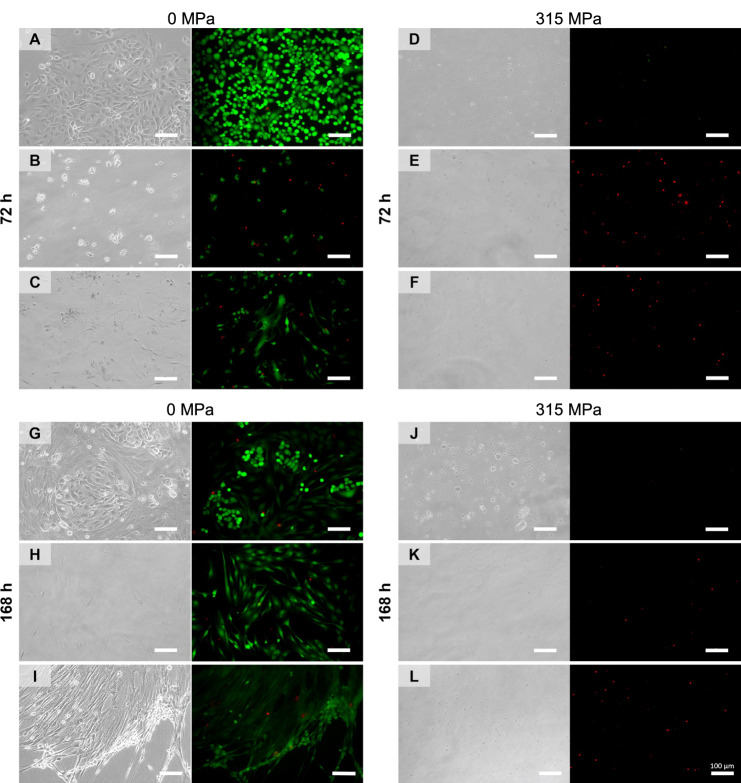
▓.

The original article has been corrected.

## Supplementary information

Supplemental Material

Figures_adjusted pressure amplitudes

HDR_pressure_curves

Pressure_curve

