# Supplementary material for: Establishing safe high hydrostatic pressure devitalization thresholds for autologous head and neck cancer vaccination and reconstruction
Source: Cell Death Discov. 2023 Oct 23;9:390. doi: 10.1038/s41420-023-01671-z (PMC10593744; doi:10.1038/s41420-023-01671-z)
Supplement: Supplementary file 1 — Supplemental Material [file 41420_2023_1671_MOESM1_ESM.docx]

**Supplemental Material**

# Supplemental figures

**Supplemental Figure 1: Flow cytometry gating strategy.** Representative results from HNSCC16 cells are shown.

**Supplemental Figure 2: Cell culture of high hydrostatic pressure treated cells.** Tumor cells were treated for 10 min at different pressure amplitudes and subsequently incubated for 24, 48 and 72 hours to observe cell growth and colony formation. Representative light microscopic images of UT-SCC-14 culture are shown.

**Supplemental Figure 3: Evidence of devitalization at 315 MPa high hydrostatic pressure. A/B** After explantation on day 14, vital autofluorescent PE/CA/PJ-15 tumor cells are shown in the matrix after 210 MPa treatment. **C/D** Treatment at 315 MPa results in complete devitalization. The PE/CA/PJ-15 cell remnants in the Matrigel matrix show no nuclei and disintegrate.

# Supplemental methods

**Cell lines**

For the experiments, cells isolated from HNSCC of the tongue (UT-SCC-14 (RRID:CVCL_7810), PE/CA-PJ15 (RRID:CVCL_2678)), the larynx (PDX-derived HNSCC16), and the hypopharynx (PDX-derived HNSCC46) were used. All experiments were performed with mycoplasma-free cells. PE/CA/PJ-15, obtained initially from the German collection of cell cultures (DSMZ; Germany), was transduced to yield stable expression of the fluorescent near-infrared protein iRFP680 (NIR). The correlation between cell death and loss of NIR fluorescence was confirmed with DAPI staining and flow cytometry. The primary tumor cells HNSCC16 and HNSCC46 were established by the research group^1^. HNSCC16 and HNSCC46 were authenticated by STR profiling (vs. respective patient tumor and PDX). All cell lines were HPV-negative.

Cells were maintained (5% CO_2_ at 37°C) in medium: DMEM/HamsF12 supplemented with 10% fetal calf serum (FCS), glutamine (2 mmol/L), and 1% Penicillin-Streptomycin (medium and antibiotics were purchased from Pan Biotech, Germany, FCS from Sigma-Aldrich, Germany, and glutamine from Biochrom, Germany). The cells were passaged with 80% to 90% confluency.

**High hydrostatic pressure treatment**

HHP treatment was performed in cryotubes (1,8 ml Nunc, Thermo Fisher Scientific, US)^2^. 5 × 10^5^ cells (*in vitro* experiments) or 10 x 10^6^ cells (CAM assay) were suspended in culture medium and added to the cryotube. The cryotubes were centrifuged at 118 x *g* for 8 min, entirely filled with culture medium, and closed free of air bubbles. The cryotubes were sealed with Parafilm M Laboratory Film (Pechiney Plastic Packaging Inc., US) and placed into water-filled, air-bubble-free centrifuge tubes. Sealed centrifuge tubes containing the samples were then placed in the glycol-filled pressure chamber of the high hydrostatic pressure device (Dustec Hochdrucktechnik GmbH, Germany). Samples were treated for 10 min or 60 min at 0, 105, 210, 315, or 420 MPa, respectively. The pressure chamber temperature was 20°C in all experiments. The cryotubes were centrifuged again after treatment, and the cell pellet was resuspended as required.

**Irradiation and ethanol treatment**

To compare the effect of different HHP amplitudes on cell viability with irradiation and ethanol treatment, autofluorescent PE/CA/PJ-15-NIR-680 cells were used. Both irradiation and ethanol treatments were performed in centrifuge tubes. Suspended in 500 µL cell culture medium, 5 × 10^5^ cells were irradiated with 70 Gy and 150 Gy (3.8 Gy/min) single radiation dose (Cs-137 γ-irradiation; IBL 637, CIS Bio-International, France). For ethanol devitalization, cells were resuspended in 70% ice-cold ethanol and incubated for 15 min. After that, ethanol was removed *via* centrifugation steps, and cells were resuspended in culture medium. Following the respective treatment, 1 x 10^5^ cells in 1 mL culture medium were cultivated in 24-well cell culture plates as triplicates until autofluorescence measurement. Three independent experiments (n = 3) were conducted.

**Crystal violet assay**

For each cell line, five independent experiments (n = 5) were performed as duplicates. Following hydrostatic pressure treatment, 1×10^5^ UT-SCC-14, HNSCC16 or HNSCC46 cells were seeded on a 24-well cell culture plate in 1.5 mL of cell culture medium and incubated at 37°C. After 72 h of incubation, the cells were stained with crystal violet: The medium was discarded, the cells were washed with 1 mL PBS, 300 μl 0.2% crystal violet (AppliChem GmbH, Germany) was added and incubated for 10 min at room temperature (RT). The crystal violet staining was removed and cells were washed twice with 1 mL PBS (Sigma-Aldrich Chemie GmbH, Germany). Next, 300 μL of 1% SDS (AppliChem GmbH, Germany) was pipetted and incubated for 10 min. In order to measure the absorbance of the samples via photometer (Glomax Multi Detection System, Promega GmbH, Germany), 100 μL of the cell suspensions were transferred to a 96-well cell culture plate. The absorbance was measured using light with a wavelength of 560 nm against the light with a reference wavelength of 450 nm.

**Apoptosis/necrosis and Calreticulin flow cytometry**

Apoptosis/necrosis of UT-SCC-14 cells was determined 24 h after HHP treatment using a flow cytometer (BD FACSVerse™, BD Pharmingen, US) in 15 independent experiments (n = 15). Cells were stained for 20 min at RT with 0.2 µM Yo-Pro 1 iodide (Ex/Em 491/509 nm; blue laser 488 nm, Thermo Fisher Scientific, US) and 20 µg/mL Propidiumiodide (PI) (Ex/Em: 535/617 nm; blue laser 488 nm, Sigma-Aldrich, Germany). PI was added shortly before flow cytometry. BD FACSuite software (BD Pharmingen, US) was used for analysis.

For Calreticulin analysis, UT-SCC-14, HNSCC16 and HNSCC46 cells were incubated for 30 min at 4°C with the polyclonal rabbit CalR primary antibody (1:50; Abgent, US). Next, cells were washed and labeled with FITC-conjugated secondary antibody (donkey anti-rabbit, 1:50; BioLegend) and incubated again for 30 min at 4°C. Control cells were stained with the secondary antibody to exclude non-specific binding of the FITC-labeled secondary antibody without using the primary antibody. Secondary antibody-positive cells were subtracted from CalR+ and secondary antibody-positive cells^1^. For each cell line, Calreticulin was examined in five independent experiments (n = 5).

**Autofluorescence measurement**

Autofluorescence of PE/CA/PJ-15-NIR-680 cells was measured after HHP treatment, irradiation, or ethanol treatment. Analysis was performed on a fluorescence multi-well plate reader (Tecan Infinite® M200, Germany) at an excitation wavelength of 680 nm (emission 720 nm, mode: fluorescence bottom reading). For estimation of cell viability, the relative fluorescence intensities of NIR680-stained untreated cells (= 0 MPa; live control) were set to 100%, and fluorescence intensities of samples were calculated. Data from n = 3 independent experiments performed in duplicates (HHP treatment) or triplicates (irradiation and ethanol) are given.

**ATP release**

For ATP release analysis, 3.5 × 10^6^ cells/mL UT-SCC-14, HNSCC16, and HNSCC46 cells in 1.5 mL were treated with HHP. Cells were incubated for 1 h at 37°C. Cell culture supernatant from seven independent experiments (n = 7) were collected and frozen immediately at −20°C. The quantitative determination of ATP was assessed using ATP Determination Kit (Molecular Probes, US) according to the manufacturer's protocol.

**Spectral flow cytometry**

Functional analysis was achieved for UT-SCC-14, HNSCC16 and HNSCC46 in three independent experiments (n = 3) by spectral flow cytometry using two in-house designed multicolor panels. Panel 1 was used to study apoptosis, necrosis, proliferation, and autophagy. Panel 2 examined viability, methuosis, and immune regulation. For this purpose, 0.5 x 10^6^cells were taken per panel and processed. All procedures were performed using staining buffer (PBS, 2 mM EDTA, 2% BSA).

Panel 1: The extracellular staining included the following markers (in 100 µL): Apotracker green (Stock solution: 80 µM, working solution: 1:100, BioLegend, US), Mitospy^TM^ Orange CMTMRos (Stock solution 1 mM, working solution: 1:200, BioLegend, US), Cyto-ID autophagy detection kit 2.0 (working solution: 1:200, Enzo Life Sciences AG, Switzerland). Cells were stained for 20 min (RT, protected from light), washed two times (350 x *g*, 5 min), followed by membrane permeabilization (True Nuclear Transcription Factor Buffer Set, BioLegend, US, True-Nuclear™ 1X Fix concentrate, 45 min, RT). Then, the True-Nuclear™ 1X Perm Buffer (BioLegend, US) was added. Cells were washed (350 x *g*, 5 min) and stained with antibodies for intracellular staining (in 100 µL True-Nuclear™ 1X Perm Buffer): V450 rat anti-histone H3 (1:40, BD Biosciences, Germany), Alexa Fluor 647 rabbit-anti-human light chain 3B (LC3B) (1:25, R&D Systems, US), Alexa Fluor 700 mouse-anti-human cleaved Poly (ADP-ribose) polymerase (PARP) (1:20, BD Biosciences, Germany), and PE/Cyanine7 mouse anti-H2A.X phospho (1:40, BioLegend, US). Staining was done for 30 min at RT. The reaction was stopped with True-Nuclear™ 1X Perm Buffer, followed by two washing steps (350 x *g*, 5 min). Cells were finally resuspended in 0.35 mL staining buffer.

Panel 2: Viability was examined using Zombie NIR^TM^ (1:5000 in 100 µL PBS, 30 min, RT, BioLegend, US). After washing two times (350 x *g*, 5 min), extracellular staining was performed for 20 min at RT in staining buffer (in 100 µL): PE-Vio 770 REA anti-human CD274 (1:50, Miltenyi Biotec, Germany), FITC mouse anti-human CD107a (LAMP-1, 1:20, BioLegend, US), and Alexa Fluor 594 rat anti-human Rab7a (1:100, BioLegend, US). Afterward, cells were washed two times, followed by membrane permeabilization (True Nuclear Transcription Factor Buffer Set, BioLegend, US, True-Nuclear™ 1X Fix concentrate, 45 min, RT). Then, the True-Nuclear™ 1X Perm Buffer was added, cells were washed (350 x *g*, 5 min) and were stained with antibodies for intracellular staining (in 100 µL True-Nuclear™ 1X Perm Buffer): APC REA anti-human B-cell lymphoma 2 (Bcl2) (Miltenyi Biotec, Germany) and Alexa Fluor 405 mouse anti-human Hif1**α** Thermo Fisher Scientific, US). Staining was done for 30 min at room temperature. The reaction was stopped with True-Nuclear™ 1X Perm Buffer, followed by two washing steps (350 x *g*, 5 min). Cells were finally resuspended in 0.35 mL staining buffer.

All measurements were completed on a spectral flow cytometer (Cytek Aurora, Cytek Biosciences, US) in the Core Facility for Cell Sorting and Cell Analysis, University Medical Center Rostock, Germany.

**Chorioallantoic membrane assay**

All animal procedures were carried out in accordance with the national guidelines and regulations (TierSchG – Tierschutzgesetz, Germany). Fertilized *Lohmann LSL* eggs (male and female) were purchased from Lohmann Deutschland GmbH & Co. KG in Germany. The eggs were incubated horizontally for six days at 37 ± 1°C and 65 ± 7%, using a Bruja Modell 84 (Bruja, Germany) with one hour scheduled rotation. At day six the CAM was dropped as described previously^3^. Then, the eggs were opened with a diameter of 1 cm using a forceps under sterile conditions (Figure 1). Sterile Nobacutis wound dressing (Nobamed, Germany) was used to seal the window and the eggs were further incubated without rotation. At day seven, the pellet of 1 x 10^6^ HHP treated or untreated cancer cells of the respective cell line (UT-SCC-14, HNSCC16, HNSCC46, PE/CA/PJ-15) were suspended in 20 µL Matrigel (Corning® Matrigel® Basement Membrane Matrix, Germany) and pipetted onto the CAM membrane. The allocation of the HHP treatment to the eggs was randomly. Embryos were inspected daily. After one week of incubation euthanasia of the embryos was performed by administration of 0.1 mL Ketamine (50 mg/mL, Hameln pharmaceuticals GmbH, Germany) on the CAM and scissors decapitation. CAM tissue, including the implantation site, was harvested for histology (UT-SCC-14, HNSCC16, HNSCC46; n = 9-21) or *ex vivo* fluorescence measurements (PE/CA/PJ-15; n = 19 for 105, 210 and 315 MPa; n = 21 for 0 MPa). 18 animals per group (total of 72) were required to achieve a power of 0.8 for α = 0.05 in an F-test.

**NightOWL LB 983 *ex vivo* imaging**

Tumors from host chicken embryos that died before the end of the experimental period were excluded from the analysis. All tumors from viable chicken embryos were analyzed.

Following euthanasia, CAMs, including the implantation site of the fluorescent PE/CA/PJ-15 cells (n = 19 for 105, 210 and 315 MPa; n = 21 for 0 MPa), were resected and placed in 24-well cell culture plates. The NightOWL LB 983 imaging system (Berthold Technologies, Germany) was used to perform measurements with a 630 nm excitation filter (center wavelength 630 nm, full-width at half-maximum 20 nm, 80%, 25.0 mm, Berthold Technologies, Germany) and a 700 nm emission filter (cwl 700 nm, fwhm 20 nm, 80%, 50.0 mm, Berthold Technologies, Germany) with 10 s illumination. Photon counts (cts/s) were analyzed using Indigo Software (Berthold Technologies, Germany).

**Histology**

CAM tissue, including the implantation site of UT-SCC-14, HNSCC16 or HNSCC46 cells (n = 9-21), was fixed in 4% phosphate-buffered formalin for three days and then embedded in paraffin. From the paraffin-embedded tissue blocks, 4 μm sections were serially cut and counterstained with H&E. In the experimental groups using non-fluorescent PDX-derived cell lines, BrdU (5-Bromo-2'-deoxyuridine; Sigma-Aldrich, US) was used for tumor tissue viability assessment: 100 µL of BrdU (20 mg/mL) was administered topically onto the CAM membrane 24 h before euthanasia. BrdU immunohistochemistry was performed using the standard high-sensitivity DAB method for S phase identification at the time of BrdU administration^4^. Mouse anti-BrdU (1:50, Agilent DAKO, US) was used as the primary antibody and goat anti-mouse HRP (1:100, Agilent DAKO, US) as the secondary antibody. A clinical pathologist analyzed histology. The pathologist was blinded to the treatment of the tumors.

**Patient-derived HNSCC tissue culture and live/dead staining**

Tissue from the HNSCC biobank^5^ (local Ethics Committee, reference number A2018-000) was obtained to study the effect on tumor tissue. Briefly, after dissection, the samples were sent to the Institute of Pathology, Rostock University Medical Center (in RT NaCl 0.9%) for an instantaneous H&E section. The pathologist removed tumor tissue for routine diagnostics and necrotic/fibrotic areas and provided macroscopically vital tumor tissue for the biobank. Fresh tumor samples from three different donors (n = 3) were treated with 0 MPa or 315 MPa for 10 min in air-free cryotubes. Treatment was performed within 90 min from resection. Next, the tumor tissue was dissociated using 2 mg/mL collagenase A (Roche Diagnostics GmbH, Germany) at 37°C under constant agitation for two hours. The cell suspension was filtered through a cell strainer (pore size: 70 μm; Greiner Bio-One GmbH, Germany), washed with PBS, and centrifuged at 118 x g for 8 min to remove the supernatant. Finally, the cells were resuspended in cell culture medium and seeded into a 12-well cell culture plate.

To assess cell morphology and viability after HHP treatment, light microscopy followed by live/dead staining was performed after 72 or 168 h, respectively. First, medium was removed, and cells were carefully washed with PBS. Then, live/dead staining was performed using the LIVE/DEAD^TM^ Viability/Cytotoxicity Kit (Invitrogen by Thermo Fischer Scientific, Life Technologies Corporation, US) according to the manufacturer's instructions. After an incubation time of 30 min at RT and under light exclusion, the tissue culture was analyzed under the fluorescence microscope (Nikon ECLIPSE TS100, Nikon GmbH, Germany) at the same position for live and dead cells. Afterward, pictures of living and dead cells were merged using ImageJ^6^.

Statistics

All values are given as mean ± standard deviation (*in vitro*) or as individual values with a median of 25% percentile/75% percentile (CAM assay). Statistical evaluation was performed using GraphPad PRISM software, version 8.0.2 (GraphPad Software, US). The criterion for significance was set at p < 0.05. After proving the assumption of normality (Shapiro-Wilk test), 2-way ANOVA with Tukey's multiple comparison post-hoc test was performed. If the normality test failed, the Kruskal-Wallis test with Dunn's multiple comparison test was performed.

1. Schoenwaelder N, Krause M, Freitag T, Schneider B, Zonnur S, Zimpfer A, Becker AS, Salewski I, Strüder DF, Lemcke H, Grosse-Thie C, Junghanss C, et al. Preclinical Head and Neck Squamous Cell Carcinoma Models for Combined Targeted Therapy Approaches. *Cancers (Basel)* 2022;14.

2. Waletzko-Hellwig J, Pohl C, Riese J, Schlosser M, Dau M, Engel N, Springer A, Bader R. Effect of High Hydrostatic Pressure on Human Trabecular Bone Regarding Cell Death and Matrix Integrity. *Front Bioeng Biotechnol* 2021;9.

3. Li M, Pathak RR, Lopez-Rivera E, Friedman SL, Aguirre-Ghiso JA, Sikora AG. The In Ovo Chick Chorioallantoic Membrane (CAM) Assay as an Efficient Xenograft Model of Hepatocellular Carcinoma. 2015;104.

4. Cameron HA. Quantitative Analysis of In Vivo Cell Proliferation. *Curr Protoc Neurosci* 2006;37.

5. Strüder D, Momper T, Irmscher N, Krause M, Liese J, Schraven S, Zimpfer A, Zonnur S, Burmeister AS, Schneider B, Frerich B, Mlynski R, et al. Establishment and characterization of patient-derived head and neck cancer models from surgical specimens and endoscopic biopsies. *Journal of Experimental and Clinical Cancer Research* 2021;40.

6. Schneider CA, Rasband WS, Eliceiri KW. NIH Image to ImageJ: 25 years of image analysis. *Nature Methods 2012 9:7* 2012;9:671–5.
