# Supplementary figures and images for: Establishing safe high hydrostatic pressure devitalization thresholds for autologous head and neck cancer vaccination and reconstruction
Source: Cell Death Discov. 2023 Oct 23;9:390. doi: 10.1038/s41420-023-01671-z (PMC10593744; doi:10.1038/s41420-023-01671-z)

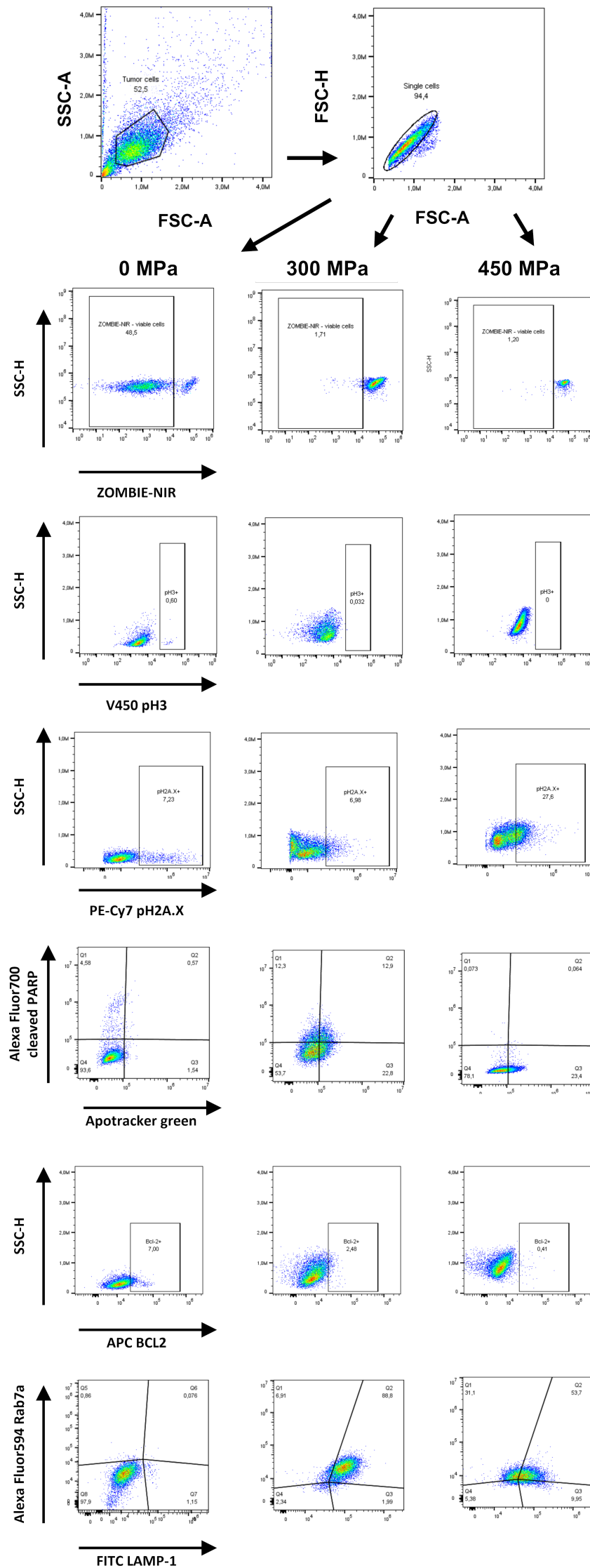

Supplement: Supplementary file 2 — Supplemental Figure 1 [file 41420_2023_1671_MOESM2_ESM.pdf]

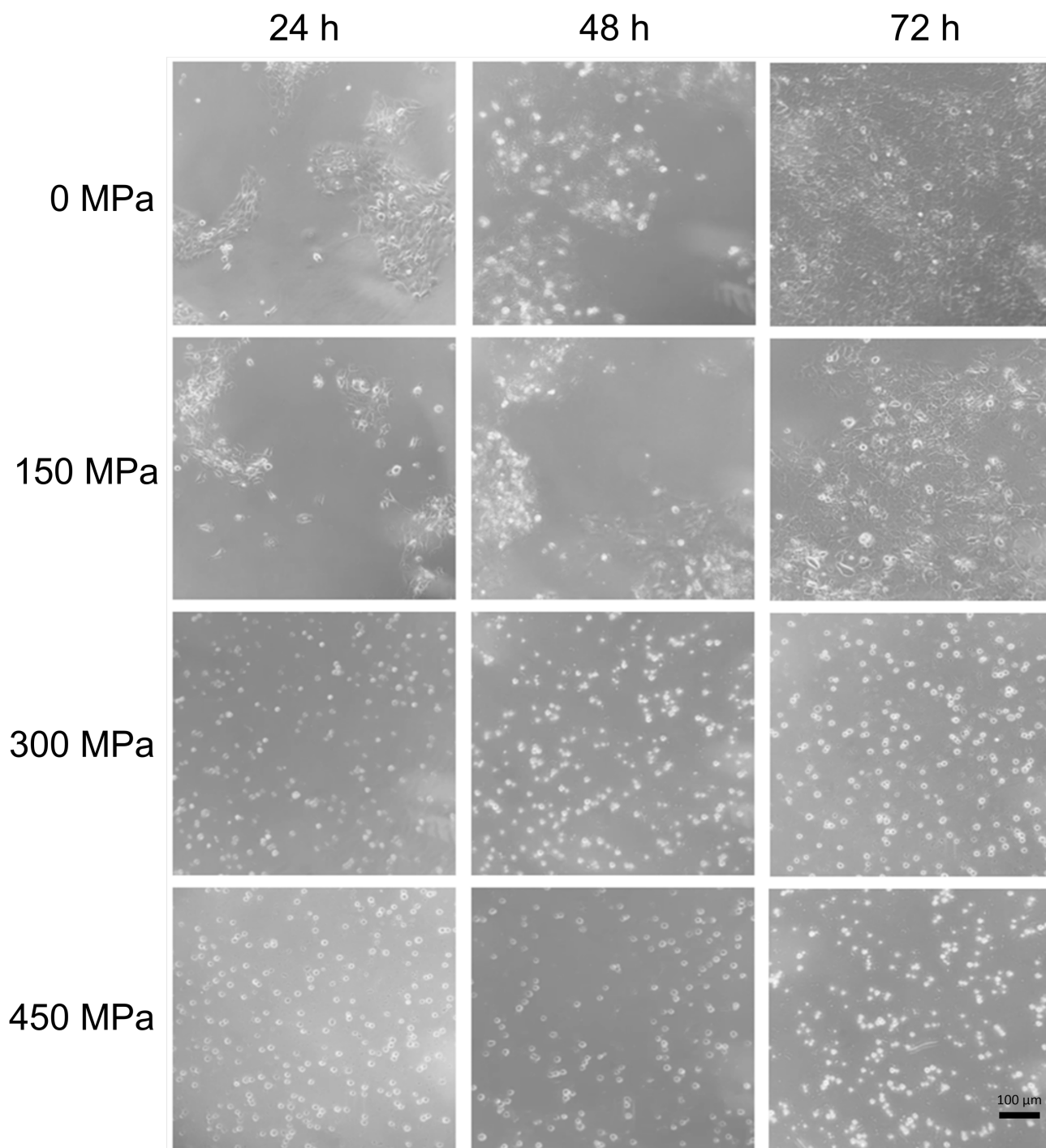

Supplement: Supplementary file 3 — Supplemental Figure 2 [file 41420_2023_1671_MOESM3_ESM.pdf]

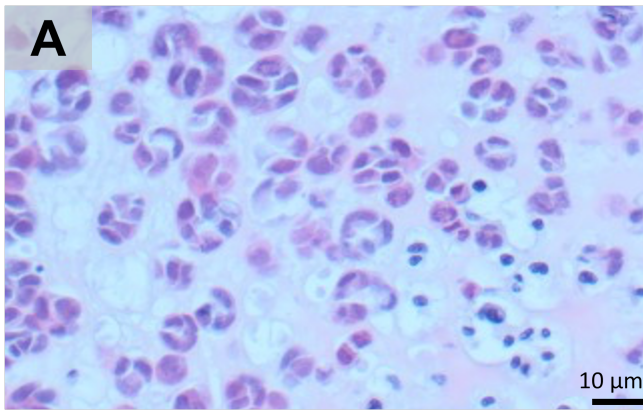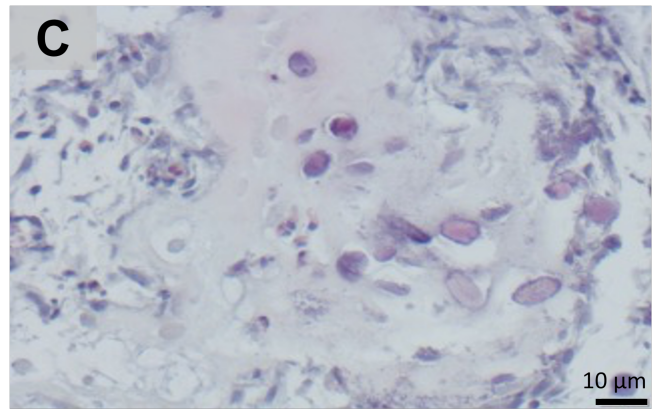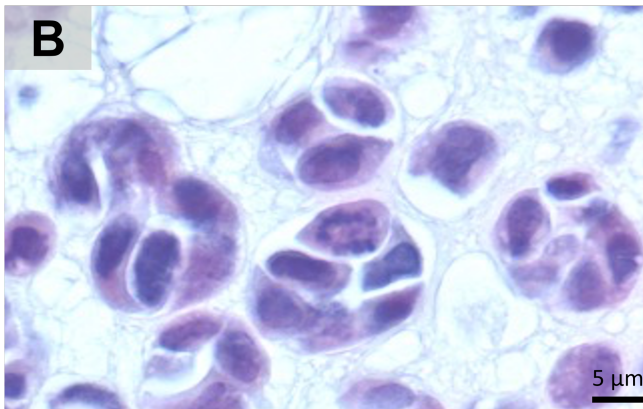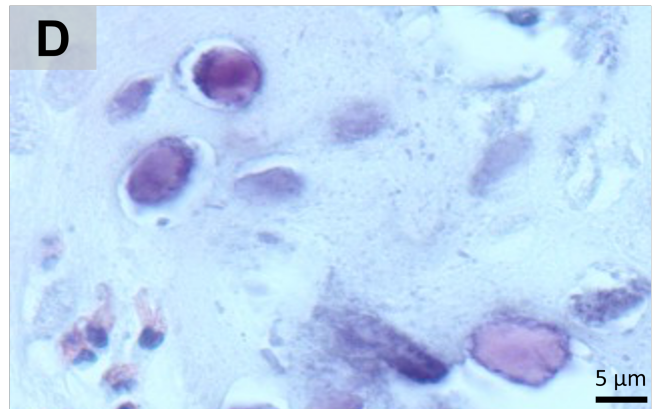

Supplement: Supplementary file 4 — Supplemental Figure 3 [file 41420_2023_1671_MOESM4_ESM.pdf]
