# Supplementary material for: Author Correction: Establishing safe high hydrostatic pressure devitalization thresholds for autologous head and neck cancer vaccination and reconstruction
Source: Cell Death Discov. 2024 Oct 15;10:437. doi: 10.1038/s41420-024-02172-3 (PMC11480040; doi:10.1038/s41420-024-02172-3)

Figure 1

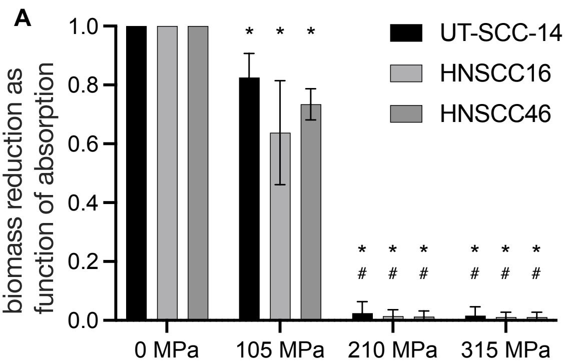

A
biomass reduction as function of absorption
UT-SCC-14
HNSCC16
HNSCC46
0 MPa
105 MPa
210 MPa
315 MPa

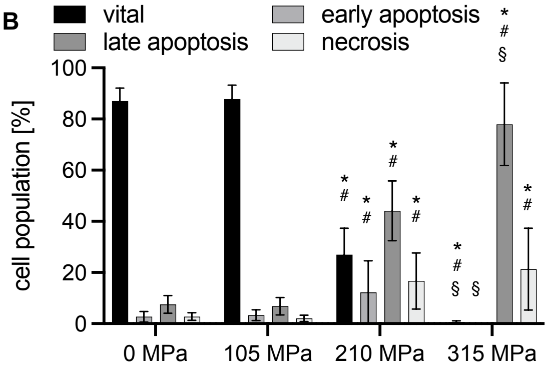

B
vital
early apoptosis
late apoptosis
necrosis
cell population [%]
0 MPa
105 MPa
210 MPa
315 MPa

Figure 2

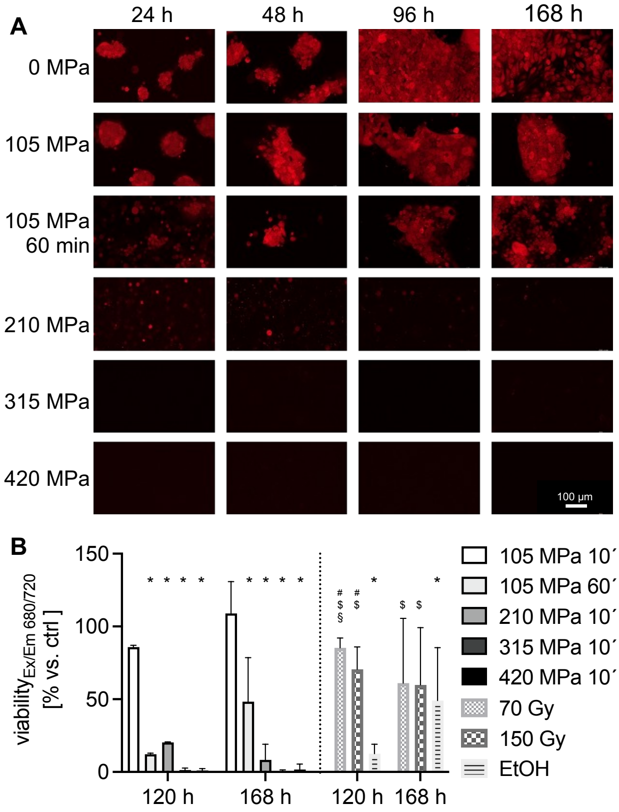

A
24 h
48 h
96 h
168 h
0 MPa
105 MPa
105 MPa 60 min
210 MPa
315 MPa
420 MPa
100 µm
B
viability$_{Ex/Em\ 680/720}$ [% vs. ctrl]
150
100
50
0
120 h
168 h
120 h
168 h
105 MPa 10´
105 MPa 60´
210 MPa 10´
315 MPa 10´
420 MPa 10´
70 Gy
150 Gy
EtOH

Figure 3

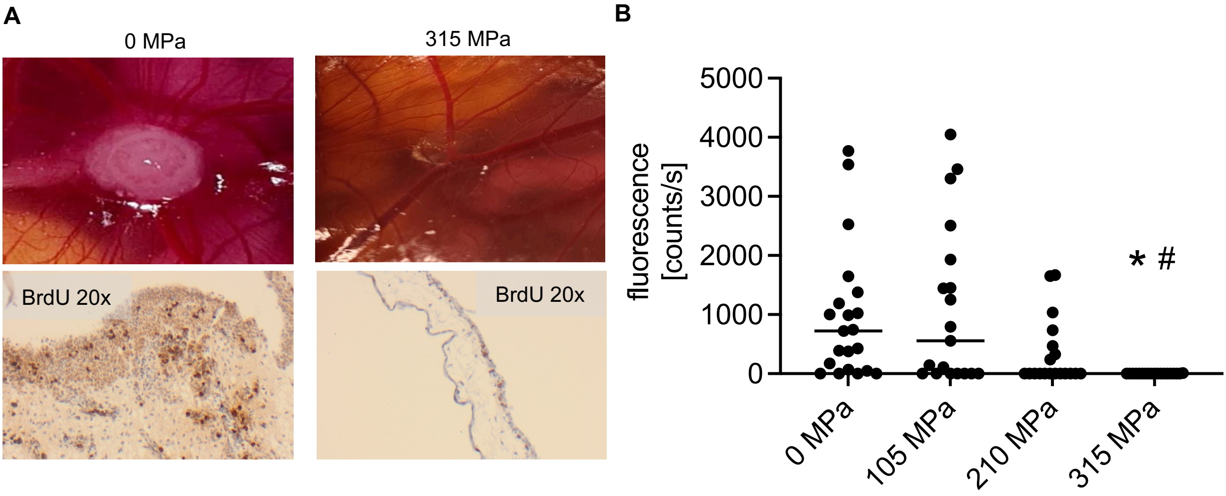

A
0 MPa
315 MPa
BrdU 20x
BrdU 20x
B
5000
4000
3000
2000
1000
0
fluorescence [counts/s]
* #
0 MPa
105 MPa
210 MPa
315 MPa

Figure 4

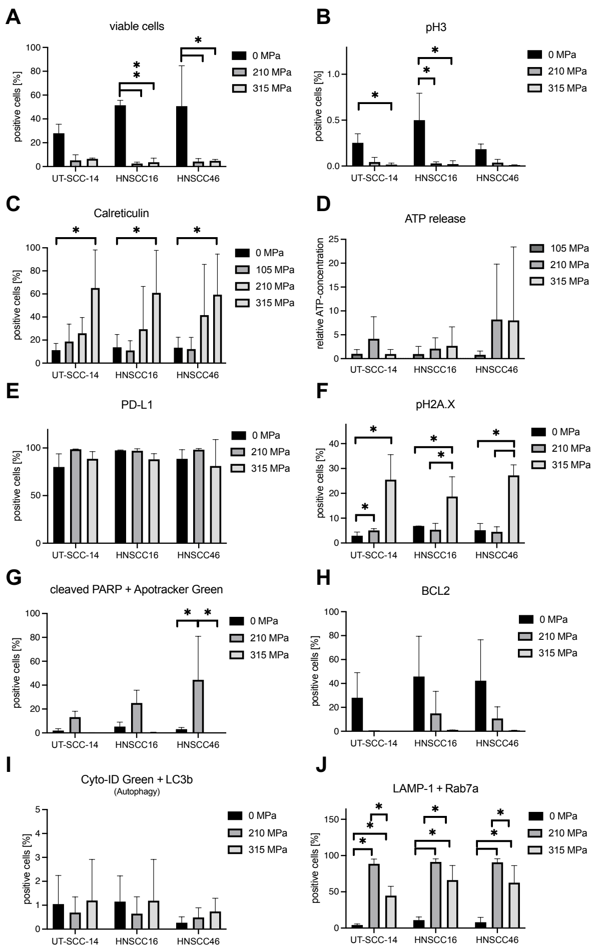

A
viable cells
positive cells [%]
0 MPa
210 MPa
315 MPa
UT-SCC-14
HNSCC16
HNSCC46
B
pH3
positive cells [%]
0 MPa
210 MPa
315 MPa
UT-SCC-14
HNSCC16
HNSCC46
C
Calreticulin
positive cells [%]
0 MPa
105 MPa
210 MPa
315 MPa
UT-SCC-14
HNSCC16
HNSCC46
D
ATP release
relative ATP-concentration
105 MPa
210 MPa
315 MPa
UT-SCC-14
HNSCC16
HNSCC46
E
PD-L1
positive cells [%]
0 MPa
210 MPa
315 MPa
UT-SCC-14
HNSCC16
HNSCC46
F
pH2A.X
positive cells [%]
0 MPa
210 MPa
315 MPa
UT-SCC-14
HNSCC16
HNSCC46
G
cleaved PARP + Apotracker Green
positive cells [%]
0 MPa
210 MPa
315 MPa
UT-SCC-14
HNSCC16
HNSCC46
H
BCL2
positive cells [%]
0 MPa
210 MPa
315 MPa
UT-SCC-14
HNSCC16
HNSCC46
I
Cyto-ID Green + LC3b
(Autophagy)
positive cells [%]
0 MPa
210 MPa
315 MPa
UT-SCC-14
HNSCC16
HNSCC46
J
LAMP-1 + Rab7a
positive cells [%]
0 MPa
210 MPa
315 MPa
UT-SCC-14
HNSCC16
HNSCC46

Figure 5

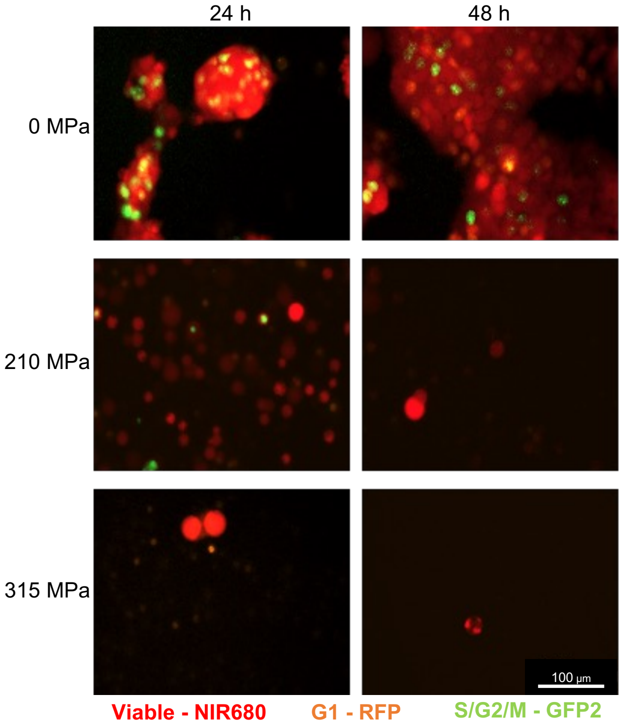

24 h
48 h
0 MPa
210 MPa
315 MPa
100 µm
Viable - NIR680
G1 - RFP
S/G2/M - GFP2

Figure 6

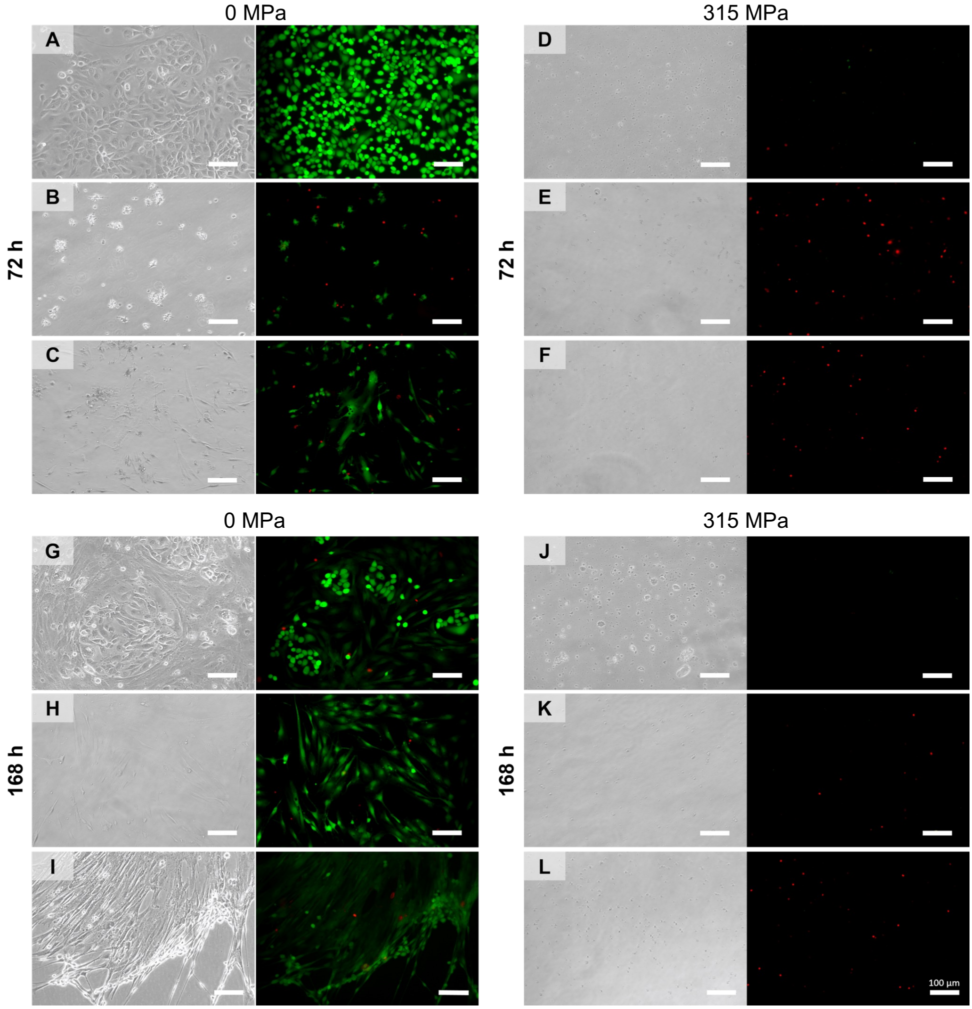

0 MPa
315 MPa
A
B
C
D
E
F
72 h
72 h
0 MPa
315 MPa
G
H
I
J
K
L
168 h
168 h
100 µm

Figure 7

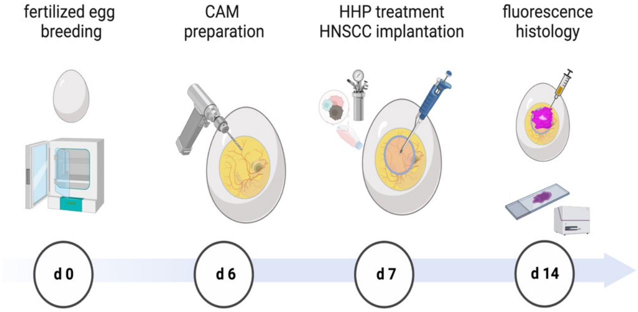
fertilized egg
breeding
CAM
preparation
HHP treatment
HNSCC implantation
fluorescence
histology
d 0
d 6
d 7
d 14

suppl. Figure 1

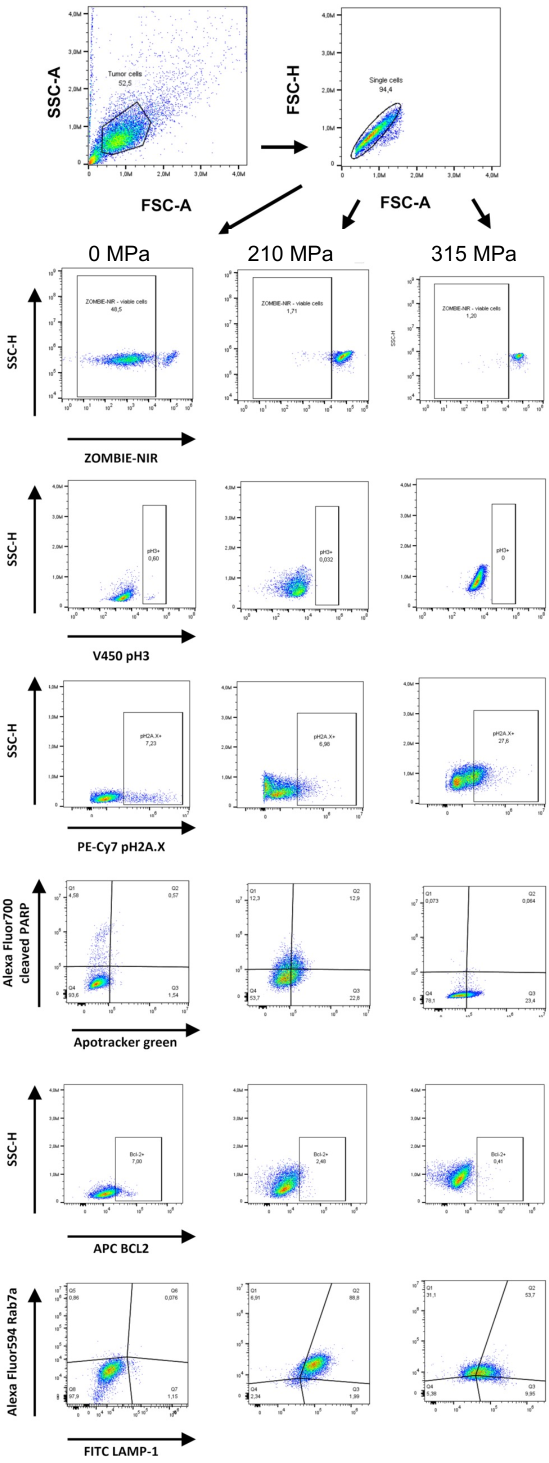

suppl. Figure 2

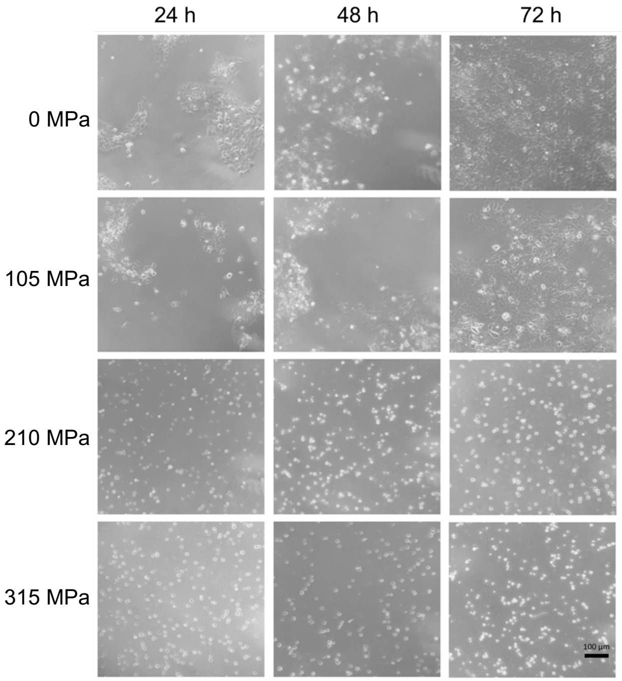

suppl. Figure 3

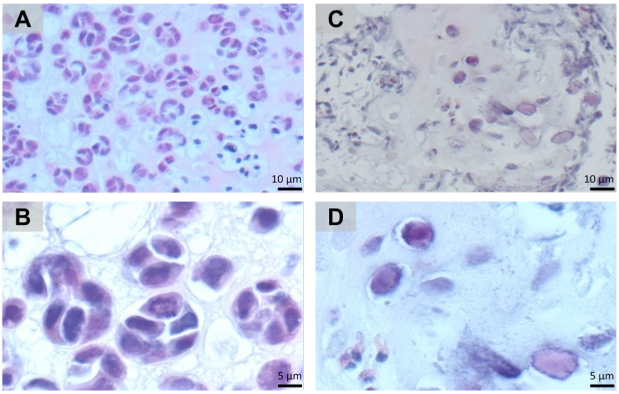

Supplement: Supplementary file 2 — Figures_adjusted pressure amplitudes [file 41420_2024_2172_MOESM2_ESM.pdf]
