# Supplementary material for: Author Correction: Establishing safe high hydrostatic pressure devitalization thresholds for autologous head and neck cancer vaccination and reconstruction
Source: Cell Death Discov. 2024 Oct 15;10:437. doi: 10.1038/s41420-024-02172-3 (PMC11480040; doi:10.1038/s41420-024-02172-3)
Supplement: Supplementary file 3 — HDR_pressure_curves [file 41420_2024_2172_MOESM3_ESM.pptx]

## Slide 1
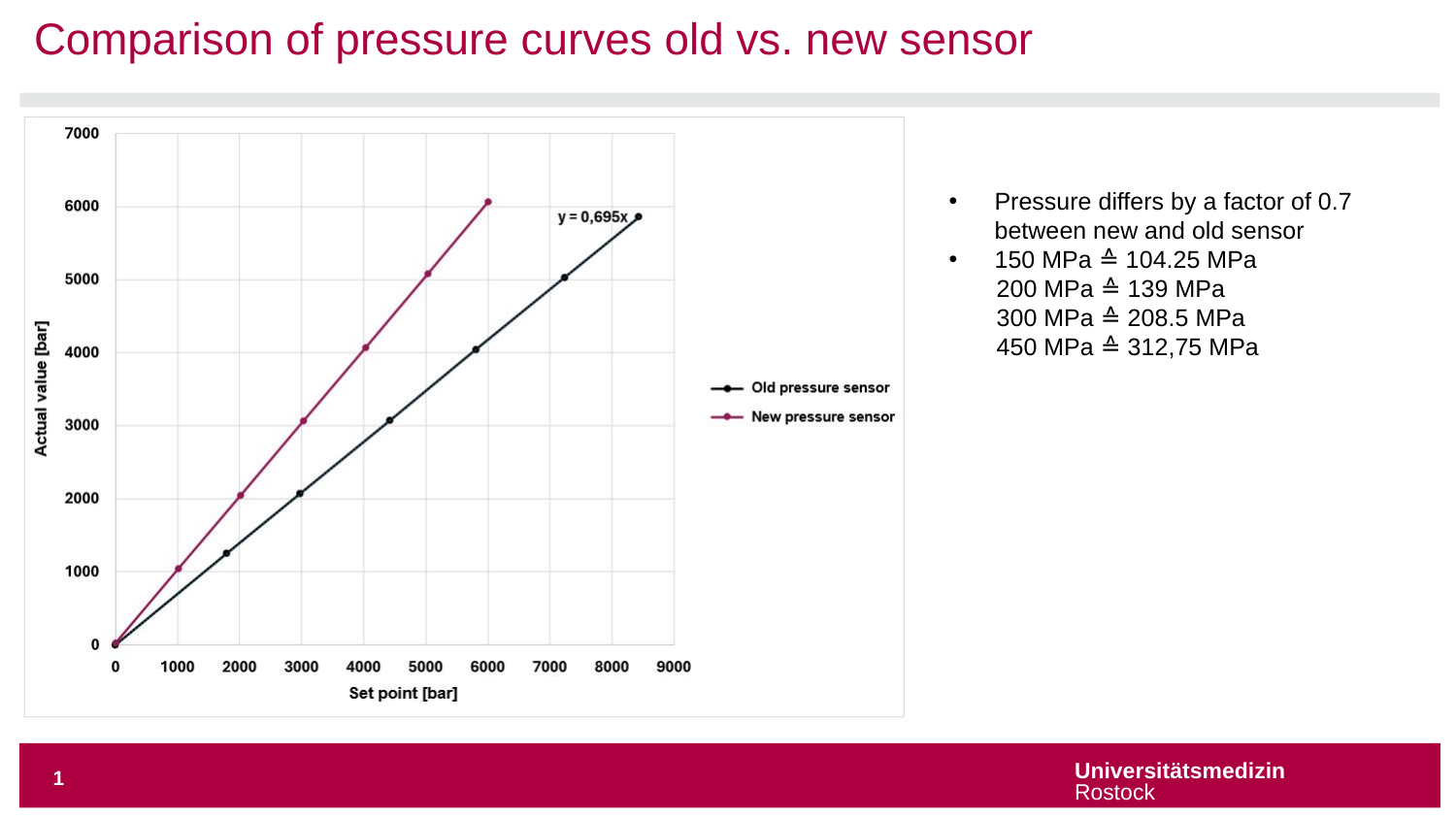

Comparison of pressure curves old vs. new sensor
Pressure differs by a factor of 0.7 between new and old sensor
150 MPa ≙ 104.25 MPa
 200 MPa ≙ 139 MPa
 300 MPa ≙ 208.5 MPa
 450 MPa ≙ 312,75 MPa
1
